# Coexpression of MEIOTIC-TOPOISOMERASE VIB-dCas9 with guide RNAs specific to a recombination hotspot is insufficient to increase crossover frequency in Arabidopsis

**DOI:** 10.1093/g3journal/jkac105

**Published:** 2022-04-29

**Authors:** Nataliya E Yelina, Daniel Holland, Sabrina Gonzalez-Jorge, Dominique Hirsz, Ziyi Yang, Ian R Henderson

**Affiliations:** Department of Plant Sciences, University of Cambridge, Cambridge CB2 3EA, UK; Department of Plant Sciences, Crop Science Centre, University of Cambridge, Cambridge CB3 0LE, UK; Department of Plant Sciences, University of Cambridge, Cambridge CB2 3EA, UK; Department of Plant Sciences, University of Cambridge, Cambridge CB2 3EA, UK; Department of Plant Sciences, University of Cambridge, Cambridge CB2 3EA, UK; Department of Plant Sciences, University of Cambridge, Cambridge CB2 3EA, UK; Department of Plant Sciences, University of Cambridge, Cambridge CB2 3EA, UK

**Keywords:** meiosis, crossover, targeted recombination, CRISPR/Cas9, MTOPVIB

## Abstract

During meiosis, homologous chromosomes pair and recombine, which can result in reciprocal crossovers that increase genetic diversity. Crossovers are unevenly distributed along eukaryote chromosomes and show repression in heterochromatin and the centromeres. Within the chromosome arms, crossovers are often concentrated in hotspots, which are typically in the kilobase range. The uneven distribution of crossovers along chromosomes, together with their low number per meiosis, creates a limitation during crop breeding, where recombination can be beneficial. Therefore, targeting crossovers to specific genome locations has the potential to accelerate crop improvement. In plants, meiotic crossovers are initiated by DNA double-strand breaks that are catalyzed by SPO11 complexes, which consist of 2 catalytic (SPO11-1 and SPO11-2) and 2 noncatalytic subunits (MTOPVIB). We used the model plant *Arabidopsis thaliana* to coexpress an MTOPVIB-dCas9 fusion protein with guide RNAs specific to the *3a* crossover hotspot. We observed that this was insufficient to significantly change meiotic crossover frequency or pattern within *3a*. We discuss the implications of our findings for targeting meiotic recombination within plant genomes.

## Introduction 

Meiosis is a specialized eukaryotic cell division where a single round of DNA replication and 2 rounds of chromosome segregation result in haploid gametes required for sexual reproduction (Villeneuve and Hillers 2001; Mercier *et al.* 2015). During prophase I of meiosis, homologous chromosomes undergo programmed recombination, which can result in reciprocal crossover (Villeneuve and Hillers 2001; Mercier *et al.* 2015). Crossovers contribute to genetic variation in progeny and result in new haplotypes, which can allow combination of useful traits in crop species (Taagen *et al.* 2020). However, recombination frequency and pattern can significantly limit breeding, as crossovers are relatively low per meiosis (typically 1–2 per chromosome) and show a highly uneven distribution (Mercier *et al.* 2015; Taagen *et al.* 2020). For example, crossovers in wheat, barley, and maize occur predominantly in the sub-telomeric regions (Higgins *et al.* 2012; Rodgers-Melnick *et al.* 2015; Darrier *et al.* 2017; Mascher *et al.* 2017), which can cause linkage drag in low-recombination regions that are under selection. Therefore, technology to increase global crossover numbers, or induce recombination at loci of choice, have the potential to substantially accelerate crop breeding.

Crossovers are initiated by double-strand breaks (DSBs) catalyzed by the conserved transesterase SPO11 (Bergerat *et al.* 1997; Keeney *et al.* 1997). SPO11 is a homolog of the archaeal topoisomerase VI catalytic A subunit that acts with noncatalytic B subunits in A_2_B_2_ heterodimers (Bouuaert and Keeney 2016; Robert *et al.* 2016). In Arabidopsis, 2 nonredundant homologs of the topoisomerase VI A subunit, SPO11-1 and SPO11-2, are required to generate meiotic DSBs (Grelon *et al.* 2001; Stacey *et al.* 2006; Hartung *et al.* 2007). The meiotic topoisomerase VIB-like subunits, MTOPVIB, interact with both SPO11-1 and SPO11-2 to catalyze meiotic DSBs in Arabidopsis and rice (Bouuaert and Keeney 2016; Fu *et al.* 2016; Vrielynck *et al.* 2016). During catalysis, SPO11 becomes covalently bound to DNA and is then removed bound to a short oligonucleotide, via endonuclease activities (Neale *et al.* 2005; Choi *et al.* 2018). The resulting DSB 5′-end is then digested by exonucleases to produce 3′ overhanging single-strand DNA (ssDNA) at each end of the DSB (Hunter 2015). Meiotic ssDNA associates with the recombinases RAD51 and DMC1 to promote ssDNA strand invasion of a homologous chromosome or a sister chromatid (Hunter 2015). Invasion of homologous DNA generates a displacement loop (D-loop), which allows extension of the 3′ ssDNA via DNA synthesis using the homologous DNA sequence as a template (Hunter 2015).

Following interhomolog or intersister strand invasion, alternative DNA repair pathways are followed during meiosis (Hunter 2015). First, the D-loop may be disassociated from the invaded template and returned to the parental chromosomes, where it is repaired as a noncrossover (Hunter 2015). If DNA synthesis occurred over a polymorphic site following inter-homolog strand invasion this may result in a gene conversion (Hunter 2015). In plants, noncrossover repair is promoted via the activity of several nonredundant proteins that include the FANCM, RECQ4A and RECQ4B helicases, FIGL1, and FLIP1 (Crismani *et al.* 2012; Girard *et al.* 2015; Séguéla-Arnaud *et al.* 2015; Fernandes *et al.* 2018). Alternatively, capture of the second resected 3′ end, followed by DNA synthesis, can form a double Holliday junction joint molecule (dHJ-JM) (Hunter 2015). The Class I pathway acts to stabilize dHJs and promotes their resolution as a crossover (Börner *et al.* 2004; Jackson *et al.* 2006; Wijeratne *et al.* 2006; Higgins *et al.* 2008, 2004; Macaisne *et al.* 2011, 2008; Chelysheva *et al.* 2012, 2007; Manhart and Alani 2016). In Arabidopsis, an estimated ∼150–250 DSBs mature into ∼10 crossovers per meiosis, with the remaining DSBs repaired as noncrossovers (Ferdous *et al.* 2012; Wijnker *et al.* 2013; Rowan *et al.* 2019). This indicates that the anti-crossover pathways mediate repair of the majority of meiotic DSBs as noncrossovers.

Chromosome structure, chromatin, and epigenetic information also exert a significant influence on meiotic recombination. At the fine-scale, meiotic DSBs and crossovers tend to cluster in narrow (kilobase) regions called hotspots (Choi and Henderson 2015). In plants and budding yeast, meiotic DSB hotspots frequently occur in nucleosome-depleted regions associated with gene control regions (Pan *et al.* 2011; He *et al.* 2017; Choi *et al.* 2018). Furthermore, RNA-directed DNA methylation and elevated nucleosome occupancy are sufficient to suppress crossovers within an Arabidopsis recombination hotspot (Yelina *et al.* 2015). Meiotic DSB formation and repair occur in the context of proteinaceous chromosome axis, which underpins meiotic chromosome architecture (Zickler and Kleckner 1999). Sister chromatids are organized as linear arrays of chromatin loops connected to the axis (Zickler and Kleckner 1999). In plants, the chromosome axis includes the HORMA domain protein ASY1 (a homolog of yeast Hop1) and its interacting partners ASY3 and ASY4, which promote DMC1-mediated interhomolog synapsis and recombination (Armstrong *et al.* 2002; Sanchez-Moran *et al.* 2007; Ferdous *et al.* 2012; Chambon *et al.* 2018). The axis also includes cohesin complexes containing the meiosis-specific REC8 α-kleisin subunit, which coheres sister chromatids and anchors the chromatin loops to the axis (Cai *et al.* 2003; Chelysheva *et al.* 2005). As prophase I progresses, the chromosomes synapse, and the synaptonemal complex is installed between them, coincident with crossover maturation (Zickler and Kleckner 1999; Hunter 2015).

Work in budding yeast has shown that tethering SPO11, or its interacting partners, using DNA binding domains is sufficient to create recombination hotspots de novo (Pecina *et al.* 2002; Acquaviva *et al.* 2013). In recent years, several technologies have emerged with the potential to tether factors of interest to specific loci. For example, translational fusions of SPO11 with zinc finger domains, TAL repeats and dCas9 have been used to target meiotic DSBs to loci of choice in budding yeast (Sarno *et al.* 2017). In this study, we coexpressed an MTOPVIB-dCas9 fusion protein with guide RNAs (gRNAs) specific to the previously characterized *3a* crossover hotspot in *Arabidopsis thaliana*. The catalytically dead *Streptococcus pyogenes* Cas9 (dCas9) carries 2 amino acid substitutions (D10A and H841A) that abolish its endonuclease activity, but do not impair its ability to bind target DNA via gRNAs (Qi *et al.* 2013). We used high-resolution crossover mapping to determine *3a* recombination frequency and distribution in MTOPVIB-dCas9 lines in the presence or absence of *3a*-specific gRNAs. We did not observe significant changes to crossover frequency or pattern with the *3a* hotspot compared to wild type. This indicates that coexpression of MTOPVIB-dCas9 with gRNAs specific to an Arabidopsis meiotic crossover hotspot is insufficient to change crossover recombination.

## Materials and methods

### Plant material and genotyping

Arabidopsis lines used in this study were Col-0, *mtopvib-1* (EDA42 line, Ws-4 accession), *mtopvib-2* (GABI_314G09, Col-0 accession) (Vrielynck *et al.* 2016), *CTL 2.10* and *CTL 5.1* (Wu *et al.* 2015), which were obtained from the Eurasian Arabidopsis Stock Centre (uNASC) and Arabidopsis Biological Resource Centre (ABRC). Plants were grown under long-day conditions (16 h light/8 h dark) at 20°C, as previously described (Yelina *et al.* 2015). Plant transformation was performed by floral dipping (Zhang *et al.* 2006). PCR genotyping of *mtopvib-1* and *mtopvib-2* was performed as described (Vrielynck *et al.* 2016). PCR genotyping of *mtopvib-2* complemented with *MTOPVIB-dCas9* transgenes was performed with MTOP-genot-compl-F and MTOP-genot-compl-R oligonucleotides. Oligonucleotides are listed in Supplementary Table 1.

### In silico gRNA design and in vitro testing

gRNAs were in silico designed using E-CRISP (Heigwer *et al.* 2014) (http://www.e-crisp.org/E-CRISP), CRISPR-P (Lei *et al.* 2014) (http://crispr.hzau.edu.cn/CRISPR2) and CRISPR-MIT (crispr.mit.edu, now obsolete) online tools. gRNAs spacer sequences and Arabidopsis genome target coordinates are listed in Supplementary Table 2. gRNA efficiencies of in silico designed gRNAs were tested in an in vitro CRISPR/Cas9 assay. Briefly, DNA fragments corresponding to *3a-P*, *3a-B*, and *3a-I* and harboring gRNA target sites were PCR-amplified using Arabidopsis genomic DNA and oligonucleotides listed in Supplementary Table 1. gRNAs were obtained by in vitro transcription using MEGAscript T7 Transcription Kit (ThermoFisher Scientific). DNA templates for in vitro transcription were PCR-amplified using pEn-Chimera vector and oligonucleotides listed in Supplementary Table 1. 300 ng of gRNA transcript was bound to a purified Cas9 protein (New England Biolabs) for 10 min at 25° C, followed by the addition of 300 ng of target DNA and incubation at 37°C for 1 h. gRNA transcripts were then cleaved by 0.3 µg/µl RNase A for 5 min at 37°C. DNA fragments were separated on a 1.5% agarose gel stained with Midori Green Advance DNA Stain (Geneflow) to visualize the presence or absence of CRISPR/Cas9-induced target DNA cleavage. gRNAs that led to target DNA cleavage in in vitro assays were used to generate constructs for Arabidopsis transformation.

### Cloning

To generate *MTOPVIB-dCas9*, a full genomic sequence of *MTOPVIB* (At1g60460) including a 2385 bp region upstream of the ATG start codon and a 294 bp region downstream of the TAG stop codon was PCR amplified with oligonucleotides MTOPVI-Prom-SalI-F and MTOPVI-Term-NotI-R and cloned between *Sal*I and *Not*I restriction endonuclease sites into pGreen0029 vector (Addgene), to yield the pGreen-gMTOPVIB construct. A *Xba*I restriction endonuclease site in the 7th intron of *MTOPVIB* was mutagenized by digesting pGreen-gMTOPVIB with *Xba*I restriction enzyme, end-filling the resulting 5′ overhang using Klenow fragment and religating to yield pGreen-gMTOPVIBΔXbaI. An *Asc*I restriction site was introduced in front of the ATG start codon by amplifying a part of the MTOPVIB promoter region with MTOPVI-*Nhe*I-F and MTOPVI-*Asc*I-R oligonucleotides and cloning the resulting fragment into *Nhe*I- and *Nco*I-digested pGreen-AscI-gMTOPVIBΔXbaI. A GGSGGS linker, a nuclear localization signal, 2 hemagglutinin (2×HA) epitope tags and *Xba*I and *Bam*HI restriction sites were introduced at the C-terminus of *MTOPVIB* upstream of the TAG stop codon by cloning a double-strand DNA fragment resulting from annealing MTOPVIB-C-HA-top and MTOPVIB-C-HA-bottom oligonucleotides into a *Pst*I-digested pGreen-AscI-gMTOPVIBΔXbaI. The resulting construct was called pGreen-gMTOPVIB-C-NLS-2×HA.

Catalytically inactive Cas9 (dCas9) was generated via PCR-site-directed mutagenesis. Briefly, Cas9 coding sequence was amplified from hSpCas9 plasmid, kindly provided by Prof Jian-Kang Zhu (Feng *et al.* 2013), in a multiplex PCR reaction using Cas9-1stMut-F, dCas9-1stMut-R, dCas9-2ndMut-F, dCas9-2ndMut-R primers and a Phusion DNA polymerase. Following PCR amplification methylated template plasmid DNA carrying wild type Cas9 was digested with *Dpn*I restriction endonuclease, PCR products carrying mutated dCas9 were ligated and transformed into *Escherichiacoli* DH5α strain. Mutations leading to D10A and H840A amino acid substitutions in the Cas9 coding sequence were confirmed by Sanger sequencing. Next, dCas9 was PCR amplified with dCas9-XbaI-F and dCas9-BamHI-R oligonucleotides and cloned into *Xba*I- and *Bam*HI- digested pGreen-gMTOPVIB-C-NLS-2HA to yield MTOPVIB-dCas9. Oligonucleotide sequences are provided in Supplementary Table 1.

To generate Cas9-gRNA-P, Cas9-gRNA-B, Cas9-gRNA-I and Cas9-non-3a-gRNA constructs, 6×(pre-tRNA-gRNA) PCR products were amplified using oligonucleotides listed in the Supplementary Table 1 and as described (Xie *et al.* 2015), digested with *Fok*I restriction endonuclease and cloned into *Bbs*I-digested pEn-Chimera vector (kindly provided by Prof Holger Puchta) behind the Arabidopsis U6 (AtU6) promoter. Fragments containing AtU6:6×(pre-tRNA-gRNA) were transferred from pEn-Chimera into binary pDe-CAS9 vector (kindly provided by Prof Holger Puchta) as described (Schiml *et al.* 2016).

To generate gRNA-P, gRNA-B, gRNA-I and non-3a-gRNA constructs, 6×(pre-tRNA-gRNA) fragments were PCR amplified as described above, digested with *Fok*I restriction endonuclease and cloned into *Bbs*I-digested pChimera vector, kindly provided by Prof Holger Puchta, behind *AtU6* promoter. Fragments containing AtU6:6×(pre-tRNA-gRNA) were excised from the resulting vectors with *Avr*II restriction endonuclease and cloned into a *Xba*I-digested binary vector pGreen0229.

### Detection of CRISPR/Cas9-induced mutations

*3a-P*, *3a-B*, and *3a-I* genetic intervals were PCR amplified from Arabidopsis T_1_ genomic DNA or wild type Col using oligonucleotides listed in Supplementary Table 1. The resulting PCR products were separated on a 1% agarose gel and stained with Midori Green Advance DNA Stain (Geneflow) to visualize full-length and deletion products. The latter were excised and extracted from an agarose gel and subject to Sanger sequencing. Deletion products that could not be resolved by agarose gels were cloned into pGem-T-easy vector (Promega) following the manufacturer’s protocol and individual clones were subject to Sanger sequencing. CRISPR/Cas9-induced mutations in *CLE10*, *CLV3*, and *GL1* destroyed *Bsu*36I, *Bsp*HI and *Dde*I restriction endonuclease sites, respectively. To detect CRISPR/Cas9-induced mutations in these genes, DNA fragments harboring gRNA target sequences were PCR-amplified and digested with the above restriction endonucleases. The resulting products were separated on 1% agarose gels and stained with Midori Green Advance DNA Stain (Geneflow). T7 endonuclease I (New England Biolabs) assays were used to detect CRISPR/Cas9-induced mutations in *CLE9*, *FWA*, and *eIF(iso)4E* as described (Pyott *et al.* 2016).

### Seed fluorescent measurement of crossovers

Seed fluorescent measurements of crossovers in *CTL 2.10* and *CTL 5.1* intervals was performed as described (Yelina *et al.* 2015), using CellProfiler (Carpenter *et al.* 2006).

### Pollen typing

Pollen typing for *3a* crossover hotspot was performed as previously described in Yelina *et al.* (2015).

### RT-PCR detection of gRNA transcripts

RNA was extracted from closed buds of 2 independent pools of F_1_ individuals used for “pollen typing” using PureZOL RNA Isolation Reagent (Bio-Rad) according to the manufacturer’s protocol. Ten micrograms of total RNA was treated with TURBO DNase (ThermoFisher Scientific) and reverse-transcribed in the presence or absence (negative control) of SuperScript IV enzyme (ThermoFisher) using random hexamer primers, according to the manufacturers’ protocols. A 1:20 dilution of the resulting cDNA was PCR-amplified using oligonucleotides listed in Supplementary Table 1. The resulting products were resolved on a 2% agarose gel stained with Midori Green Advance DNA Stain (Geneflow).

### Kompetitive Allele-Specific PCR (KASP) Assay

Arabidopsis genomic DNA was extracted as described in Edwards *et al.* (1991). Kompetitive Allele-Specific PCR (KASP) Assay was performed following the manufacturer’s protocol using KASP master mix (LGC Biosearch Technologies) and oligonucleotides listed in Supplementary Table 1. Reactions were run on a CFX real-time PCR system (Bio-Rad), allele discrimination was performed using the manufacturer’s software.

### ChIP-qPCR

ChIP was performed as described (Lambing *et al.* 2020) using ∼10 g of closed flower buds as starting material and 40 µl of anti-HA antibody (#ab9110 Abcam) per genotype. qPCR was performed using Luna Universal qPCR Master Mix (New England Biolabs).

## Results

### *MTOPVIB-dCas9* functionally complements *mtopvib*

**Fig. 1.**
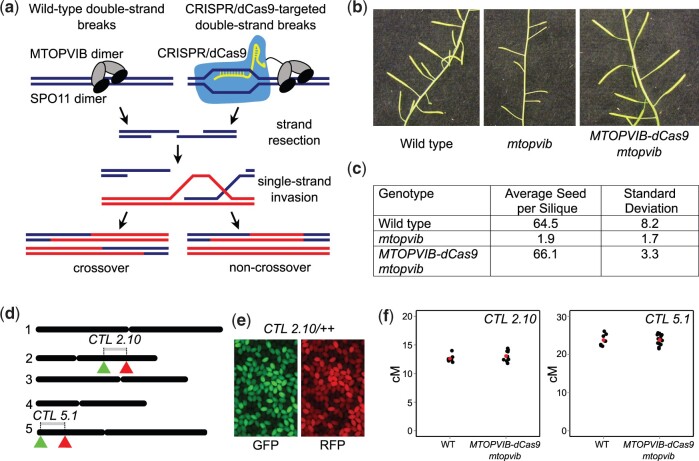
Complementation of Arabidopsis *mtopvib* with MTOPVIB fused to catalytically inactive Cas9 (MTOPVIB-dCas9). a) Wild type and synthetic pathways to generate meiotic double-strand breaks. Homologous chromosomes are shown as red and blue lines, MTOPVIB as gray ovals, SPO11 homologs as black ovals, CRISPR/dCas9 shown in blue, guide RNA paired to a genomic locus in yellow. b) Arabidopsis inflorescences showing long fruit (siliques) in wild type and complementing lines (MTOPVIB-dCas9 in *mtopvib* background) and short fruit (siliques) in *mtopvib*. c) Average seed count per silique and standard deviation for each genotype. d) Seed-based reporter systems to measure crossovers in 2 tester intervals, interstitial *CTL 2.10* on chromosome 2 and sub-telomeric *CTL 5.1* on chromosome 5. Five Arabidopsis chromosomes are shown as black lines, reporter transgenes, *eGFP*, and *dsRED*, represented by green and red triangles, respectively. e) Fluorescent micrographs showing *CTL 2.10* (*GFP RFP/++)* seed using green or red fluorescent filters. f) Genetic distances of *CTL 2.10* and *CTL 5.1* in wild type and *MTOPVIB-dCas9 mtopvib*. Each black dot represents crossover frequency in an individual plant, red dots denote mean crossover frequencies. Whitney–Mann test showed that mean crossover frequencies in *CTL 2.10* and *CTL 5.1* were not significantly different between wild type and complementing lines (*P* values of 0.54 and 0.68, respectively).

Meiotic DSBs catalyzed by Arabidopsis SPO11-1, SPO11-2 and MTOPVIB are essential to initiate crossover formation (Fig. 1a) (Grelon *et al.* 2001; Stacey *et al.* 2006; Hartung *et al.* 2007; Vrielynck *et al.* 2016). We translationally fused *Streptococcus pyogenes* dCas9 to the C-terminus of Arabidopsis MTOPVIB and asked whether the fusion protein complements the function of wild-type *MTOPVIB* (Fig. 1, b–f). We expressed an *MTOPVIB-dCas9* translational fusion gene under the control of the endogenous *MTOPVIB* promoter and terminator, in an *mtopvib-2* (hereafter, *mtopvib*) null mutant background (Vrielynck *et al.* 2016). Crossovers physically link homologous chromosomes during prophase I of meiosis ensuring balanced chromosome segregation. Therefore, an absence of meiotic DSBs and crossovers in *mtopvib* (or *spo11-1* and *spo11-2*) mutants leads to unbalanced, aneuploid gametes and almost complete sterility (Fig. 1, b and c) (Grelon *et al.* 2001; Stacey *et al.* 2006; Hartung *et al.* 2007; Vrielynck *et al.* 2016). For example, we observed an average of ∼1.9 ± 1.7 seeds per fruit (silique) in *mtopvib*, compared to ∼64.5 ± 8.2 in the wild type (2-tailed *t*-test, *P < *0.00001) (Fig. 1, b and c; Supplementary Table 3). In contrast, *MTOPVIB-dCas9 mtopvib* shows an average seed set of 66.1 ± 3.3 seeds per silique that was not significantly different from wild type (2-tailed *t*-test, *P = *0.63) (Fig. 1, b and c, Supplementary Table 3), indicating that the MTOPVIB-dCas9 fusion protein functionally complements *mtopvib*. To further confirm this, we used fluorescent crossover reporters to measure genetic distances (crossover frequency) in 2 intervals, *CTL2.10*, an interstitial region on chromosome 2, and *CTL5.1*, a sub-telomeric region on chromosome 5 (Wu *et al.* 2015), in *MTOPVIB-dCas9 mtopvib* and wild type (Fig. 1, d–f and Supplementary Tables 4 and 5). We found that mean genetic distances in these intervals were not significantly different between wild type and *MTOPVIB-dCas9 mtopvib* (Whitney-Mann tests, *P = *0.54 and 0.68, respectively). This further demonstrates that the MTOPVIB-dCas9 fusion protein is functional and supports a normal level of crossover.

### Selecting *3a* meiotic crossover hotspot as a target locus for de novo crossovers

**Fig. 2.**
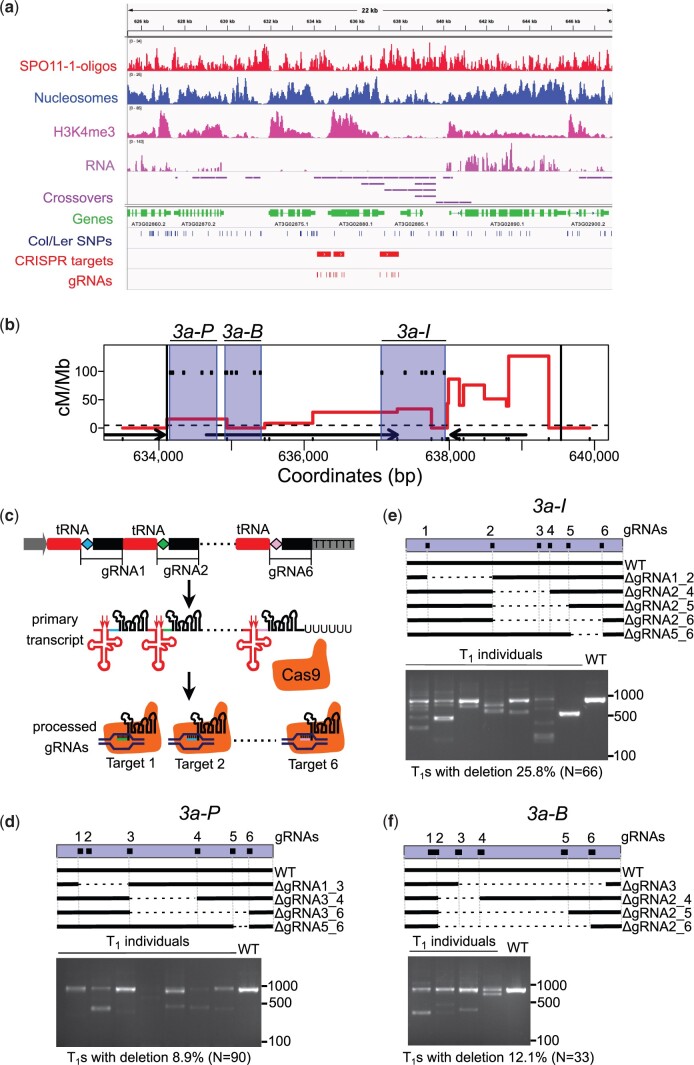
Testing gRNAs targeting *3a* meiotic recombination hotspot via a catalytically active Cas9. a) Histograms for the chromosome 3 sub-telomeric region showing library size normalized coverage values for SPO11-1-oligonucleotides (red), nucleosome occupancy (blue, MNase-seq), H3K4me3 (pink, ChIP-seq), RNA-seq (lilac) and crossovers (purple). Positions of CRISPR target regions are shown as red rectangles and individual gRNA target loci as red ticks. TAIR 10 gene annotations are shown in green and single nucleotide polymorphisms between Col and Ler as blue ticks. b) *3a* crossover profile, red line (centimorgans per megabase, cM/Mb), in Col/Ws *MTOPVIB-dCas9 mtopvib* F1s. Black vertical lines delineate borders of the *3a* hotspot, ticks on the *x*-axis represent polymorphisms between Col and Ws. Black arrows represent genes, dashed horizontal line—male chromosome 3 average crossover frequency. Six gRNAs were designed to target each of the 3 regions within *3a*, *3a-P*, *3a-B*, and *3a-I*, shaded in blue. gRNA target sites are shown as black ticks within the blue shaded areas. c) Multiplexing 6 gRNAs via endogenous tRNA-processing system. Schematic representation of a gRNA-tRNA transgene containing tandemly arranged tRNAs and gRNAs. Pol III promoter—grey arrow, terminator—grey rectangle, guide RNA-specific spacers are shown as diamonds of different colors (blue, green, or pink), conserved gRNA scaffold shown as black rectangles, tRNA as red rectangles. The primary transcript is cleaved by endogenous RNase P and RNase Z (red arrows) to release mature tRNA (red cloverleaf structure). Processed mature gRNAs guide catalytically active Cas9 (orange) to specific targets. gRNAs 3-5 and their targets are not shown. d) CRISPR/Cas9-induced deletions in *3a-P*. *3a-P* is shown as blue rectangle, 6 gRNAs as black squares. Wild-type and deleted regions within *3a-P* are shown by black and dashed lines, respectively. Midori-green-stained agarose gel image shows PCR-amplified *3a-P* in wild type (WT) and representative individual T1s. Lower than wild type molecular weight products result from CRISRP/Cas9-mediated deletions in *3a-P*. Percentage of T1s with CRISRP/Cas9 induced deletions and the total number of T1s analyzed are indicated under the agarose gel image. e) As in (d) but for *3a-I* region. f) As in (d) but for *3a-B* region.

We chose to induce de novo crossovers in the *3a* crossover hotspot (Yelina *et al.* 2012, 2015; Choi *et al.* 2013), which is located in a sub-telomeric region of chromosome 3 (Fig. 2, a and b, Supplementary Table 6). *3a* is a 5.8 kb region with a genetic distance of ∼0.2 cM (33.3 cM/Mb) in F_1_ hybrids between Col-0 (hereafter, Col) and Ler-0 (hereafter, Ler) *Arabidopsis thaliana* accessions (Yelina *et al.* 2012, 2015; Choi *et al.* 2013). Crossover rates within *3a* are up to ∼17 times higher than the chromosome 3 average of 4.77 cM/Mb in male meiosis (Giraut *et al.* 2011). We chose the *3a* hotspot first because data from budding yeast showed that tethering SPO11 to recombination hotspots leads to additional DSB formation (Sarno *et al.* 2017), whereas tethering to recombination “cold” regions exhibited variable and less predictable stimulations (Robine *et al.* 2007; Pan *et al.* 2011; Panizza *et al.* 2011; Ito *et al.* 2014; Sarno *et al.* 2017). Second, *3a* crossover levels are below their potential maximum in wild type, as we have previously shown a ∼40% increase in *3a* crossover frequency in *met1* mutants (Yelina *et al.* 2012). Third, we have an established “pollen typing” assay that allows us to measure *3a* crossover rates and fine-map crossover positions in this region (Yelina *et al.* 2012, 2015; Choi *et al.* 2013). We designed gRNAs to target 3 regions within *3a*: (1) the At3g02880 promoter and 5′ end (hereafter, *3a-P*), (2) the At3g02880 gene body (hereafter, *3a-B*), and (3) the intergenic region between At3g02880 and At3g02885 (hereafter, *3a-I*) (Fig. 2, a and b, Supplementary Tables 2 and 6). Notably, these regions vary in nucleosome occupancy, which is a major determinant of meiotic DSB levels in Arabidopsis (Fig. 2a) (Choi *et al.* 2018).

### Testing gRNA gene editing efficiency using catalytically active Cas9

We designed a total of 18 gRNAs within *3a*, 6 targeting each of the 3 regions within *3a* (*3a-P*, *3a-B*, and *3a-I*), with the rationale that multiple gRNAs may increase the efficiency of targeting, compared to a single gRNA (Fig. 2, a–f and Supplementary Table 2) (Chavez *et al.* 2016; Sarno *et al.* 2017). To simultaneously express 6 gRNAs using 1 T-DNA construct, we used an approach successfully employed in Arabidopsis, rice and wheat, where multiple gRNAs are expressed as part of a tRNA-gRNA synthetic transcript (Xie *et al.* 2015; Wang *et al.* 2018; Hui *et al.* 2019). We designed and assembled 6 tandemly arranged pre-tRNA-gRNA modules differing only in the sequences of gRNA spacers (Fig. 2c). pre-tRNA-gRNA synthetic transcripts mimic native tRNA-snoRNA43 transcripts in plants, allowing RNase P and Z to cleave the tRNA structure and release mature gRNAs (Fig. 2c) (Phizicky and Hopper 2010; Xie *et al.* 2015). We tested the efficiencies of in silico designed gRNAs by coexpressing 6×(pre-tRNA-gRNA) cassettes targeting *3a-P*, *3a-B*, or *3a-I* with catalytically active *S. pyogenes* Cas9 in wild type Col (Fig. 2, d–f) (Schiml *et al.* 2016). We transformed *Cas9-gRNA-P*, *Cas9-gRNA-B*, and *Cas9-gRNA-I* constructs into Arabidopsis and analyzed gene editing events within *3a* in T_1_ progeny. T_1_ individuals are usually chimeric due to somatic gene editing events (Hui *et al.* 2019). Using PCR amplification across the gRNA target sites, we observed deletions in the respective target regions in 8.9%, 12.1%, and 25.8% of T_1_ progeny of *Cas9-gRNA-P*, *Cas9-gRNA-B*, and *Cas9-gRNA-I*-transformed plants (Fig. 2, d–f and Supplementary Table 7). Sanger sequencing of these PCR products confirmed deletions associated with 17 of the 18 tested gRNAs (Fig. 2, d–f and Supplementary Figs. 1, 2, and Supplementary Table 7).

In addition, we generated a synthetic 6×(pre-tRNA-gRNA) construct to express previously reported gRNAs targeting 6 Arabidopsis genes (At1g69320, At1g26600, At2g27250, At3g27920, At4g25530, and At5g35620) outside *3a* to use as a negative control (Pyott *et al.* 2016; Hahn *et al.* 2017; Yamaguchi *et al.* 2017; Gallego-Bartolomé *et al.* 2018). We refer to this construct as *Cas9*-*non-3a-gRNA*. We transformed *Cas9*-*non-3a-gRNA* into wild-type Col and observed gene editing events in the target genes in ∼4–50% of the T_1_ progeny (Supplementary Fig. 3 and Supplementary Table 8). In summary, we obtained a set of gRNAs robustly targeting the Arabidopsis genome within and outside the *3a* crossover hotspot.

### Analysis of *3a* crossovers in the presence of MTOPVIB-dCas9 and gRNAs

**Fig. 3.**
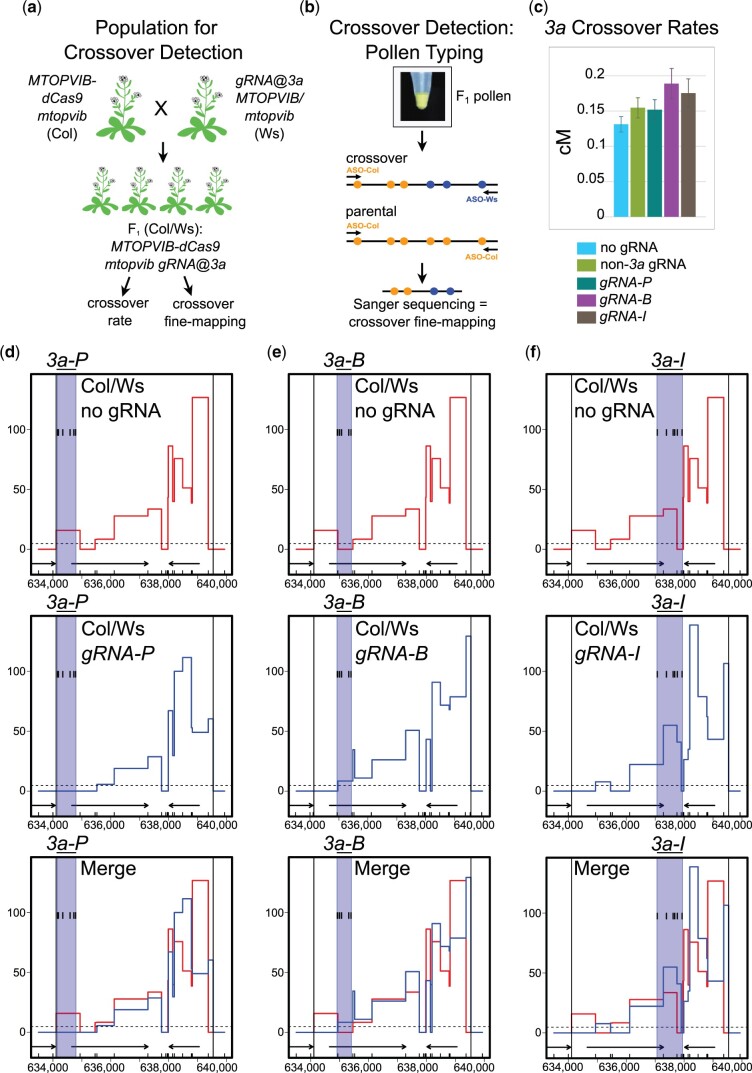
3a crossover rates in targeted and wild type F1 hybrids. a) Generation of F1 populations for fine-scale crossover analysis via “pollen typing.” Col *mtopvib* lines complemented with *MTOPVIB-dCas9* were crossed to Ws *MTOPVIB/mtopvib* carrying a guide RNA transgene targeting *3a* crossover hotspot (gRNA@3a). The resulting Col/Ws F1 populations were selected for the presence of *MTOPVIB-dCas9* transgene, absence of wild-type *MTOPVIB* (*mtopvib*) and presence of gRNA transgene. A similar crossing scheme was performed for negative controls, “no gRNA” and “non-3a gRNA,” not shown. b) Schematic representation of “pollen typing”. Genomic DNA extracted from F1 pollen is subject to PCR amplification with allele-specific oligonucleotides (ASO) to determine the concentration of recombinant crossover molecules relative to parentals. Recombinant molecules are then subject to Sanger sequencing to determine crossover distribution within the hotspot. DNA molecules shown as black lines. Yellow and blue circles represent Col- and Ws-specific polymorphisms, respectively. c) *3a* crossover frequencies in centimorgans (cM) measured by pollen typing in 5 different F1 populations. Error bars represent standard deviation. d) Fine-scale *3a* crossover profiles in the presence or absence of *gRNA-P* gRNAs targeting *3a-P*. *3a* recombination rates in centimorgans per megabase (cM/Mb) were analyzed by pollen typing. Black vertical lines delineate borders of *3a* hotspot, ticks on the *x*-axis represent polymorphisms between Col and Ws. Black arrows represent genes, dashed horizontal line—male chromosome 3 average crossover frequency. Blue shaded area (*3a-P*) marks guide RNA target region with black ticks representing individual guide RNA target sites. Recombination rates in Col/Ws *MTOPVIB-dCas9 mtopvib* F1s in the absence of guide RNAs are shown in red and in the presence of *gRNA-P* gRNAs—in blue. e) As in (d), but for *3a-B*. f) As in (d) but for *3a-I*.

We next asked whether combining *MTOPVIB-dCas9* and gRNAs that target *3a-P*, *3a-B*, or *3a-I* would affect 3a crossover rates or distribution. Crossover detection at *3a* hotspot relies on the segregation of DNA sequence polymorphisms through meiosis (Yelina *et al.* 2012, 2015; Choi *et al.* 2013). As *MTOPVIB-dCas9 mtopvib* lines were in the Col background, we generated transgenic lines expressing gRNAs in a different Arabidopsis accession, Ws-4 (hereafter, Ws), that was also heterozygous for a *mtopvib* mutation (*mtopvib-1*) (Vrielynck *et al.* 2016). The resulting lines, each of which carried a 6×(pre-tRNA-gRNA) transgene targeting *3a-P*, *3a-B*, or *3a-I*, or 6 Arabidopsis genes outside *3a*, were called *gRNA-P*, *gRNA-B*, *gRNA-I* or *non-3a gRNA*. Ws had a single nucleotide polymorphism (SNP) in position -3 relative to PAM in a target site of one of the *gRNA-B*-specific gRNAs, the remaining 17 *3a*-specific gRNAs we used targeted regions without any polymorphisms between Col and Ws. We crossed *MTOPVIB-dCas9 mtopvib* in the Col background to *gRNA-P*, *gRNA-B*, and *gRNA-I* lines in the Ws background. We then identified F_1_ progeny that were *mtopvib* null mutants and that expressed both the *MTOPVIB-dCas9* and gRNA transgenes (Fig. 3a, Supplementary Fig. S4). We also crossed Col *MTOPVIB-dCas9 mtopvib* to Ws *MTOPVIB/mtopvib* to generate a “no gRNA” F_1_ population as a negative control.

Given the *3a* crossover rate of ∼0.2 cM, to characterize ∼100 crossover events, it is necessary to assay ∼50,000 meioses. To achieve this we employed “pollen typing,” which is a PCR-based assay used to amplify and quantify crossover and parental molecules from pollen DNA (Fig. 3b) (Drouaud and Mézard 2011; Choi *et al.* 2017). To perform pollen typing, we first extract genomic DNA from F_1_ pollen. The pollen DNA contains *3a* parental and crossover molecules distinguishable by DNA sequence polymorphisms between the accessions (Col and Ws) (Fig. 3b). We perform 2 rounds of allele-specific PCR, using primers that anneal to polymorphic sites, to specifically amplify crossover or parental molecules (Fig. 3b). For quantification, we use titration where pollen template DNA is diluted until approximately half of PCR amplification reactions are negative (Drouaud and Mézard 2011; Choi *et al.* 2017). We also Sanger sequenced the amplified crossover molecules to map internal crossover locations within the *3a* hotspot (Drouaud and Mézard 2011; Choi *et al.* 2017).

We employed pollen typing to measure *3a* crossover frequency (genetic distance) and observed ∼0.13–0.15 cM in Col/Ws F_1_s in the absence of gRNAs (Fig. 3c and Supplementary Table 9). We observed no significant crossover rate changes in F_1_ populations expressing *gRNA-B*, *gRNA-I*, or *gRNA-P* (0.189 cM, chi-square test, *P = *0.44, 0.175 cM, *P = *0.64 and 0.152 cM, *P = *0.96, respectively), compared to negative controls (0.155 and 0.131 cM) (Fig. 3c and Supplementary Table 9). We Sanger sequenced between 77 and 90 crossover molecules for each F_1_ population and found that crossover profiles were very similar in the presence or absence of gRNAs targeting *3a* (Fig. 3, d–f and Supplementary Table 10). In all cases, we observed lower crossover frequencies at the telomere-proximal end and higher crossover frequencies toward the centromere-proximal end of *3a* (Fig. 3, d–f and Supplementary Table 10). These data indicate that targeting MTOPVIB-dCas9 to *3a* does not have a strong effect on crossover rate or distribution.

**Fig. 4.**
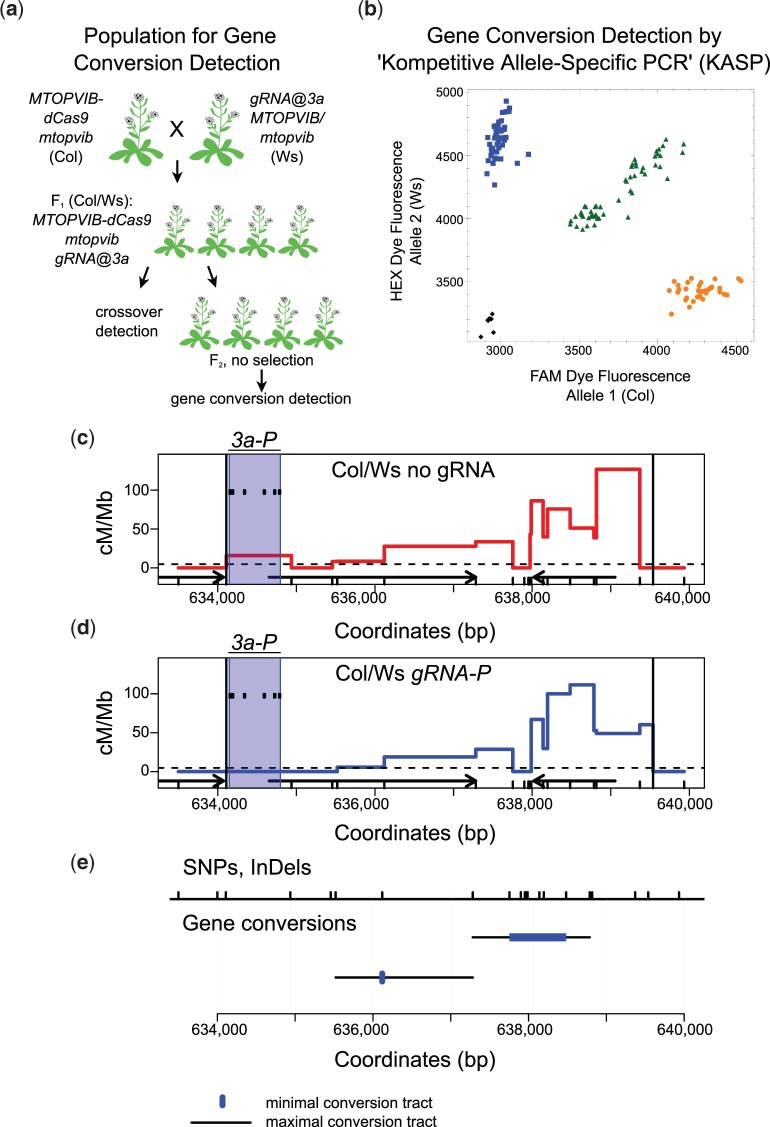
Gene conversions in *3a* (a) Generation of F2 populations for gene conversion detection via Kompetitive Allele-Specific PCR (KASP). b) An example plot showing allele discrimination via KASP assay for 1 single nucleotide polymorphism (SNP) between Col and Ws. Each dot represents an F2 individual. Different colors—yellow, blue, green, and black—represent Col, Ws, heterozygous and a “no DNA” control, respectively. c) *3a* fine-scale crossover profile, red line, in centimorgan per megabase (cM/Mb) in Col/Ws *MTOPVIB-dCas9 mtopvib* F1 population. Black vertical lines delineate borders of *3a* hotspot, ticks on the *x*-axis represent polymorphisms between Col and Ws. Black arrows represent genes, dashed horizontal line—male chromosome 3 average crossover frequency. *3a-P* target region is shaded in blue and positions of individual guide RNAs are shown as black ticks. d) As in (c) but in the presence of *gRNA-P* gRNAs. e) Gene conversion events detected in Col/Ws *MTOPVIB-dCas9 gRNA-P* F2 population. DNA sequence polymorphisms (SNPs and InDels) are shown as black ticks at the top of the plot. Maximum and minimum gene conversion tracts shown as black lines and blue rectangles, respectively.

In Arabidopsis, a minority of meiotic DSBs (∼5%–10%) are repaired as crossovers (Copenhaver *et al.* 1998; Giraut *et al.* 2011; Chelysheva *et al.* 2012, 2007; Salomé *et al.* 2012; Serrentino and Borde 2012; Choi *et al.* 2018). Noncrossovers are an alternative outcome of meiotic DSB repair and, therefore, we asked whether targeting MTOPVIB-dCas9 to *3a* could result in increased noncrossovers, measured via gene conversion. To detect gene conversion we used 4 F_2_ populations, which were the progeny of Col/Ws F_1_ expressing either *gRNA-P*, *gRNA-B* or *gRNA-I*, or “no gRNA” as a negative control (Fig. 4a). We employed a Kompetitive Allele-Specific PCR (KASP) assay to distinguish between SNP alleles (Fig. 4b). We designed 12 KASP assays to distinguish between Col and Ws alleles within, as well as up to 5.2 kb up- and 4.5 kb downstream of *3a*. Physical distances between the markers used for KASP assays ranged from 0.7 to 2.0 kb, with an average of 1.3 kb (Supplementary Table 11). Initially, we used ∼83–96 F_2_ individuals for each of the 4 F_2_ populations and detected 2 gene conversion events in the F_2_ population expressing *gRNA-P* and none in the other 3 populations, including the “no gRNA” negative control. Next we increased *gRNA-P* and “no gRNA” F_2_ population sizes to the total of ∼470 individuals each but did not detect any additional gene conversion events (Fig. 4, c–e and Supplementary Table 12). Therefore, we did not observe any gene conversion events in *gRNA-B*, *gRNA-I* or the negative control, but observed 2 gene conversions out of 469 F_2_ individuals in *gRNA-P*. Next we performed a combination of Sanger sequencing and KASP assays at additional SNPs to confirm our initial results and to determine gene conversion tract lengths. We found that one of the gene conversion events, which had a Ws to Col to Ws genotype, occurred in a low polymorphism region and its tract length could vary from a minimum of 1 to a maximum of 1,763 bp. The other gene conversion event, which had a Col to Ws to Col genotype, occurred in a region more densely covered with polymorphisms. Its conversion tract could vary from a minimum of 729 to a maximum of 1,503 bp (Fig. 4e, Supplementary Table 13). Lack of additional crossover or gene conversion events at *3a* is consistent with the lack of increased levels of MTOPVIB-dCas9 enrichment at *3a* in the presence of *3a*-specific gRNAs that we observed via ChIP-qPCR analysis (Supplementary Fig. 5).

## Discussion

In this study, we aimed to introduce de novo crossovers in a meiotic crossover hotspot *3a* by targeting Arabidopsis MTOPVIB, which is essential for initiation of meiotic DSBs, to *3a* via CRISPR. We confirmed that the MTOPVIB-dCas9 translational fusion functionally complements the *mtopvib* mutant. We also confirmed the functionality of the gRNAs we used via catalytically active Cas9 mutagenesis at target loci. The *3a* crossover hotspot is a 5.8 kb sub-telomeric interval with recombination up to ∼20 times higher than the chromosome average (Yelina *et al.* 2012, 2015; Choi *et al.* 2013). We chose *3a* hotspot as a target first because *3a* crossover rates are amenable to manipulation. For example, we have previously shown that recruitment of heterochromatic features, including DNA methylation and H3K9me2, reduces *3a* crossover rates ∼2–3 times (Yelina *et al.* 2015). *3a* crossover rates are also not at their maximum level in wild type, as genome-wide loss of CG context DNA methylation in *met1* results in a ∼40% increase in *3a* crossover frequency (Yelina *et al.* 2012). Second, because studies in budding yeast have shown that tethering SPO11 to recombination hot spots leads to a more robust de novo DSB induction compared to targeting SPO11 to DSB cold spots (Ito *et al.* 2014; Sarno *et al.* 2017). Mapping of SPO11-1-oligonucleotides in Arabidopsis has revealed that they accumulate at higher levels in nucleosome-free regions (Choi *et al.* 2018). Therefore, we chose gRNAs to target 3 locations within *3a* that vary in the nucleosome occupancy levels. We observe very modest and statistically insignificant increases to crossover frequencies and a very similar crossover topology within *3a* when MTOPVIB-dCas9 is expressed in the presence of *gRNA-P*, *gRNA-B*, or *gRNA-I* compared to the negative controls.

To explain these results, it is important to note that although DSBs and crossovers correlate positively at the chromosome-scale, there are also regions where the relationship is less strong (He *et al.* 2017; Choi *et al.* 2018). Meiotic DSB repair in Arabidopsis is a multistep process with only ∼5–10% of DSBs typically maturing into crossovers (Copenhaver *et al.* 1998; Giraut *et al.* 2011; Chelysheva *et al.* 2012, 2007; Salomé *et al.* 2012; Serrentino and Borde 2012; Choi *et al.* 2018). This is in contrast to budding yeast where over a half of meiotic DSBs are repaired as crossovers (Mancera *et al.* 2008; Pan *et al.* 2011). This could explain why tethering of SPO11 to DSB hotpots in yeast robustly increases recombination (Sarno *et al.* 2017). Another explanation for our results is that any additional DSBs at the *3a* locus would be repaired via noncrossover pathways. Counter to this, we also did not measure a significant increase in gene conversions in *MTOPVIB-Cas9*. Specifically, we observed 2 gene conversion events at a frequency of ∼0.21% per SNP each following targeting of MTOPVIB-dCas9 by *gRNA-B* only. Both gene conversion events occurred 1.3–3 kb downstream of the *gRNA-B* target site and did not overlap with each other or *gRNA-B*. The gene conversion frequency we observed is similar to the previously reported Arabidopsis gene conversion frequencies of 0.017–0.55% per SNP at a meiotic crossover hotspot (Drouaud *et al.* 2013). However, it is important to note that noncrossovers are only detectable when they lead to gene conversions. In Arabidopsis, detectable gene conversion rates are extremely low, with an average of 1.7 per meiosis and are around 100–150 base pairs in length (Lu *et al.* 2012; Wijnker *et al.* 2013). The *3a* SNPs measured for gene conversion are spaced 0.7 to 2 kb apart. Hence, it is possible that many gene conversions that occur within these intervals would not be detectable. Alternatively, the lack of increased gene conversion frequency upon coexpression of MTOPVIB-dCas9 with *3a*-specific gRNAs may imply that meiotic DSB repair occurs using the sister chromatid as a template (Cifuentes *et al.* 2013; Yao *et al.* 2020).

Efficiency of MTOPVIB-dCas9 recruitment to the *3a* crossover hotspot could be another possible reason to explain our results. In wild type, SPO11-1-MTOPVIB are recruited to the *3a* crossover hotspot (Choi *et al.* 2018). We hypothesize that in our attempt to tether MTOPVIB-dCas9 we potentially create a competition between the CRISPR-mediated tethering of MTOPVIB-dCas9 and endogenous SPO11-MTOPVIB binding at the *3a* target locus. The observed lack of increase in *3a* crossovers upon coexpression of MTOPVIB-dCas9 with *3a*-specific gRNAs may be either because CRISPR-mediated targeting is weaker than the intrinsic ability of SPO11-MTOPVIB complexes to bind *3a* or because, unlike *3a* crossovers, *3a* DSBs and/or SPO11-MTOPVIB complexes, are at their maximum, preventing recruitment of additional MTOPVIB-dCas9. CRISPR/dCas-mediated targeting efficiencies could also vary between different cell types. Although U6 snRNAs are expressed in meiocytes (Yang *et al.* 2011; Barra *et al.* 2021), and we show that our *3a*-specific gRNAs driven by the *AtU6-26* promoter are expressed in Arabidopsis closed buds that contain meiotic cells, we cannot rule out that *AtU6-26* promoter is less active in meiocytes compared to other cell types resulting in lower-than-expected efficiency of MTOPVIB-dCas9 recruitment to *3a*.

Targeted crossovers remain a sought-after technology in plant genetics, as they can potentially help overcome linkage drag between deleterious and beneficial traits and address a significant bottleneck in crop breeding (Reynolds *et al.* 2021). Recently, CRISPR/Cas-mediated chromosome engineering in somatic cells has provided an alternative strategy to target homologous recombination (Hayut *et al.* 2017; Kouranov *et al.* 2022). Two recent studies have shown that DSBs induced by Cas9 in somatic cells of F_1_ hybrids can be repaired via homologous recombination resulting in targeted somatic crossovers (Hayut *et al.* 2017; Kouranov *et al.* 2022). These crossovers can be transmitted through the germline to the next generation (Hayut *et al.* 2017; Kouranov *et al.* 2022). A further study also addressed crossover suppression that can occur in hybrids due to an inversion of a chromosomal fragment in one of the parents (Schmidt *et al.* 2020). Here, CRISPR/Cas9 was used to flip an inversion of a chromosome fragment in Arabidopsis somatic cells of one of the parents, which in the context of a hybrid was able to restore meiotic crossovers (Schmidt *et al.* 2020).

In conclusion, we show that coexpression of MTOPVIB-dCas9 with gRNAs specific to the *3a* Arabidopsis meiotic recombination hotspot leads to no significant changes in crossover frequency or pattern. This highlights the complexity of plant meiotic recombination control and possible caveats in CRISPR/dCas9-mediated targeting of plant meiotic recombination factors. We propose that combined recruitment of crossover designation factors and modulation of DSB repair pathways to favor crossovers as well as optimization of recruitment technologies in meiocytes could be alternative strategies to boost plant meiotic crossovers in specific genome locations.

## Data availability

All plasmids, reagents, and Arabidopsis transgenic lines generated in this study are available upon request. Supplementary Tables 1–13 contain raw data used for fertility and crossover frequency scoring, as well as genomic positions of *3a* crossover hotspot, guide RNAs, SNPs, and oligonucleotides used in this study. Supplementary Figs. 1–3 contain CRISPR/Cas9 gene editing analysis, Supplementary Fig. 4 contains confirmation of gRNA expression, Supplementary Fig. 5 contains ChIP-qPCR analysis.

Supplemental material is available at *G3* online.

## Supplementary Material

jkac105_Supplementary_Figure_S1

jkac105_Supplementary_Figure_S2

jkac105_Supplementary_Figure_S3

jkac105_Supplementary_Figure_S4

jkac105_Supplementary_Figure_S5

jkac105_Supplementary_Tables

jkac105_Supplementary_Figure_Legends
