# Supplementary material for: Coexpression of MEIOTIC-TOPOISOMERASE VIB-dCas9 with guide RNAs specific to a recombination hotspot is insufficient to increase crossover frequency in Arabidopsis
Source: G3 (Bethesda). 2022 Apr 29;12(7):jkac105. doi: 10.1093/g3journal/jkac105 (PMC9258527; doi:10.1093/g3journal/jkac105)
Supplement: jkac105_Supplementary_Figure_S1 [file jkac105_supplementary_figure_s1.pdf]

3a-P

ATGAATACCACTTCTTAAGTTCTCTGTTGACCATTTCTATTACCTTCCTTTCAAATTGATACAACAAATACATAAAAAAA

gRNA-P-1 gRNA-P-2
AAAAAAAAATGTCTATGGTACAA ACTTATATCAGAAATAAAAA CGGTGTCGGAGAGGCTAATCTTTGTTTGTCAA CGGTA
GCTCAGACACATTTGACAGACCTGGCATGTCTTTGGTCACACTCAACCTGCAAAAAAAAAAAACATAAAAGAAACAAAAC
gRNA-P-3
GATAACGACCATTAATTTTTTAATTAGACCTAATTTATTGATCTAAT ACAAAAGAAAGGAAAAACAGAGGTCGTTTAAAC
GTCTACAAAAAACATTACTGCAGTGATTTATTGTCATTTATGTAACGCAAATATAGTTGACTCTGATAAAATTGTTACAA
CTAGTATTCATAAGTCATAACCTTATAATTAATTAATTATCTTTCTGTAAATTAGCTGTCAAATTAAATACATAAGATTC
gRNA-P-4
TCTTTTAACTTTTTTGTCAAAAAGGAAATATTCCAATTAGTAACTTTTCTTTTTTT CCTTTTTCTCTGCAATTATGACC A
TTTTCTCTCTCTTAGCCGGCTCTAGAGAGAGAAGAAAGACAGAGCTGAAACTCTTGTAAACCAAAAAACACAAAAGTCTC
gRNA-P-5
TTTGTTCAAACACTCGTTTACGCGTAG CCGAAATAAATTTTCCAAAAACC CATATCC GAAAAAGTCGATATATTTATTGA
gRNA-P-6
ATTTTTGGAA CCAAGGAGACGAAGAATTTACAC GGTTTTTGAAGAAGAAGAAGAAGATGAAGTATAAGCGTAAGCTAAGT
CTCTCTGTTGTCTTCCTCTTTGTCTTCTATCTCGCTGCGGTGACTTCAGATCTAGAGT

436-bp deletion

gRNA-P-3 gRNA-P-6
Wt GATCTAAT ACAAAAGAAAGGAAAAACAGAGGTCG.......GGAACCAAGGAGACGAAGAATTTACAC GGTTTTTG
Mut GATCTAAT ACAAAAGAAAGGAA--GACGAAGAATTTACAC GGTTTTTG

238-bp deletion

gRNA-P-3 gRNA-P-4
Wt GATCTAAT ACAAAAGAAAGGAAAAACAGAGGTCG....TTTTCCTTTTTCTCTGCAATTATGACC ATTTTCTC
Mut GATCTAAT ACAAAAGAAAGGAAAAA--TCTCTGCAATTATGACC ATTTTCTC

423-bp deletion

gRNA-P-1 gRNA-P-4
Wt GTCTATGGTACAA ACTTATATCAGAAATAAAAA CGGTGT......TTTCCTTTTTCTCTGCAATTATGACC ATTTTCTCTCT
Mut GTCTATGGTACAA ACTTATATCAGAAATA --- TCTCTGCAATTATGACC ATTTTCTCTCT

63-bp deletion

gRNA-P-5 gRNA-P-6
Wt CGCGTAG CCGAAATAAATTTTCCAAAAACC CATA .........GGAACCAAGGAGACGAAGAATTTACAC GGTTTTTGA
Mut CGCGTAG CCGAAA---AGACGAAGAATTTACAC GGTTTTTGA

3a-B

gRNA-B-1 and gRNA-B-2
TGTCTTCTATCTCGCTGCGGTGACTTCAGATCTAGAGTCTGA CCG**GAGAGCTTTACTCGCTGTTC**GTAACAGTGTCCGT
gRNA-B-3
GGCCGTCCTTTGCTATGGAACATGAGTGCTTCTTCTCCTTGTAATTG GCACGGAGTCCACTGCGATG CGGGTCGGGTC
gRNA-B-4
ACGGCTCTCCGATTACCCGGATCTGGTTTATTCGGT TCTTTACCAATCGGTGGTAT TGGTAATCTAACCCAGCTTAAGA
CTCTTTCTCTCCGGTTCAATTCTCTCTCTGGTCCTATCCCTTCGGATTTCTCCAACCTTGTTCTCCTCCGTTACTTGTATCTT
CAAGGTAATGCCTTTTCCGGTGAGATTCCGTCGCTTCTCTTCACGCTTCCGAGCATAATCAGAATCAATCTAGGGGAGA
gRNA-B-5
ATAAATTCTCGGGTCGGATCCCGGATAATGTCAATTCTGCGAC CCGGTTGGTTACTCTGTATT TGGAGAGGAATCAAC
gRNA-B-6
TCTCTGGTCCGATCCCTGAGATCACGCTTCCTCTTCAGCAATTCAAT GTTTCTTCTAATCAGTTAAA CGGGTCTATTCCG
AGTTCGTTGTCGTCTTGGCCTCGAACTGCTTTTGAAGGTAACACTCTCTGTGGGAAGCCTTTAGACACTTGTGAGGCAG

464-bp deletion

gRNA-B-1 and gRNA-B-2 gRNA-B-6
Wt TGACCG**GAGAGCTTTACTCGCTGTTC**GTAACAGTGTCCGTGGCCGT…TTCAAT GTTTCTTCTAATCAGTTAAA CGGGTCT
Mut TGACCG**GAGAGCTTTACTCGCTGTTC** GTAACAGTGT---AAACGGGTCT

208-bp deletion

gRNA-B-1 and gRNA-B-2 gRNA-B-5
Wt TGACCG**GAGAGCTTTACTCGCTGTTC**GTAACAGTGTCCGTGGCCGT…GCGACCCGGTTGGTTACTCTGTATT TGGAGA
Mut TGACCG**GAGAGCTTTACTCGCTGTTC**GTAACAGTGT--TTGGAGA

161-bp deletion

gRNA-B-1 and gRNA-B-2 gRNA-B-4
Wt TGACCG**GAGAGCTTTACTCGCTGTTC**GTAACAGTGTCCGTGGCCGT…TCGGTTCTTTACCAATCGGTGGTAT TGGTAAT
Mut TGACCG**GAG**--GTAT TGGTAAT

500-bp deletion

gRNA-B-3
Wt TTCTCCTTGTAATTG GCACGGAGTCCACTGCGATG CGGGTCG....TGTGGGAAGCCTTTAGACACTTGTGAGGCAG
Mut TTCTCCTTGTAATTG GCACGGAGTCC---TTAGACACTTGTGAGGCAG

Supplemental Figure S1
