# Supplementary material for: Coexpression of MEIOTIC-TOPOISOMERASE VIB-dCas9 with guide RNAs specific to a recombination hotspot is insufficient to increase crossover frequency in Arabidopsis
Source: G3 (Bethesda). 2022 Apr 29;12(7):jkac105. doi: 10.1093/g3journal/jkac105 (PMC9258527; doi:10.1093/g3journal/jkac105)
Supplement: jkac105_Supplementary_Figure_S2 [file jkac105_supplementary_figure_s2.pdf]

*3a-I*

gRNA-I-1
TGGGGTCAAGTTGCAATTTCTTGAATAGTGTCGAC CCCACTTCCCGCCTTTTTTCTACTTCTTCTTCTTTGTTCTTGTTC
TAGTTTGTTCGTTAATTCTTGAGCCTCAAATTGAGATGAGACCTATCAGTTTCTTTTTTTCTTTCTTTCTACTTTTCTTT
ATTTCCAAAGTTTTAAAAATGTCTATAAAACTATTGCTTTTGCTAATTTTTGTATAAATATATAGTTGATTGCGTGTGAA

TAAATTGGTTTTTGTTTTCTTCTTTACACATCTTATCCCAGAATCCTAGTCAACGATTCAGCCAAAAAACAATGTCAACG
gRNA-I-2
AGTTTTATTTTTTATCGCCGTCCAAAATTTTGAGCCAAA AACAAAGTCAACGACAAAAGTGGTCATGAATATCG TCTCAC
GATTGACTGACAAATACTTCACGATTTGATTAGATATGAAGTTTTATAATGTGAAGTTGGTAACCACTAACTAATAACCC
TACTTCACAGGAGAAAGAAAAAACATTTTAGATATGAAAAAACAACATTGACCCAGAAAGATATATATTTGCTTAGAGAT
gRNA-I-3
TTTGTATGAAAGAATTACTAGTGCTGATAA ACTATTTAGTTCAAGCTTAA AGGGAAATCGATTAGATAATTT TTCGAATT
gRNA-I-4
ATTTGAAGAAAACATATTGTGATC TGGACTTATAATTTTTCATGTGATATACCATTTACACCGAGTCATGGCAGTTGCC
gRNA-I-5
ACAACTTTATCTCAT GTTCCAACTCGTACCAGACT GGGTACATATGCACATGGGGTTCAATAAATTTATTAGTGGGATAC
TCTTTGACATAAAACACTATTAGGAAAAAATGGAATTAAAACCTATTCATAAATT CATATAATTACGCACGTAAATAATG
gRNA-I-6
TTTTTCACAGTAATATTAGA ATTTGACCTTAGCAAGTTGA TGGTTTTTAAACCGTGGAAGCAAATAGAAC

**376-bp deletion**

gRNA-I-2 gRNA-I-5
Wt GCCAAAAACAAAGTCAACGACAAAAGTGGTC..........................CTCATGTTCCAACTCGTACCAGACTGGGTACATATGC
Mut GCCAAAAACAAAGTCAACGACAA------------------------------------------------------------------------------------------ACTGGGTACATATGC

**8-bp insertion**

gRNA-I-5
Wt GCAGTTGCCACAACTTTATCTCAT GTTCCAACTCGTACCAG-----------------ACTGGGTACAT
Mut GCAGTTGCCACAACTTTATCTCAT GTTCCAACTCGTACCAG**TTGCCACA**ACTGGGTACAT

**285-bp deletion**

gRNA-I-2 gRNA-I-4
Wt GCCAAAAACAAAGTCAACGACAAAAGTGGTC...........ATTTGAAGAAAACATATTGTGATC TGGACTTATAA
Mut GCCAAAAACAAAGTCAACGACAA-------------------------------------------------------------------ATCTGGACTTATAA

**335 bp deletion and 1 bp insertion**

gRNA-I-1 gRNA-I-2
Wt TCGACCCCACTTCCCGCCTTTTTTCTACTTCTTCTTC........GCCAAAAACAAAGTCAACGACAAAAGTGGTCAT
Mut TCGACCCCACT----------------------------------------------------------------------------------------------------------AAAGTGGTCAT

**164 bp deletion and 1 bp insertion**

gRNA-I-5 gRNA-I-6
Wt TCTCATGTTCCAACTCGTACCAGACTGGGTACAT... ..........TAGAATTTGACCTTAGCAAGTTGA TGGTTTTTAAACC
Mut TCTCATGTTCCAACTCGTACCAG-----------------------------------------------------------------------------TTGATGGTTTTTAAACC

**541 bp deletion**

gRNA-I-2 gRNA-I-6
Wt CAAAAACAAAGTCAACGACAAAAGTGGTCAAGAATTTGACCTTAGCAAGTTGA TGGTTTT
Mut CAAAAACAAAGTCAACGACAA------------------------------------------------------------TGATGGTTTT

Supplemental Figure S2
