# Supplementary figures and images for: Coexpression of MEIOTIC-TOPOISOMERASE VIB-dCas9 with guide RNAs specific to a recombination hotspot is insufficient to increase crossover frequency in Arabidopsis
Source: G3 (Bethesda). 2022 Apr 29;12(7):jkac105. doi: 10.1093/g3journal/jkac105 (PMC9258527; doi:10.1093/g3journal/jkac105)

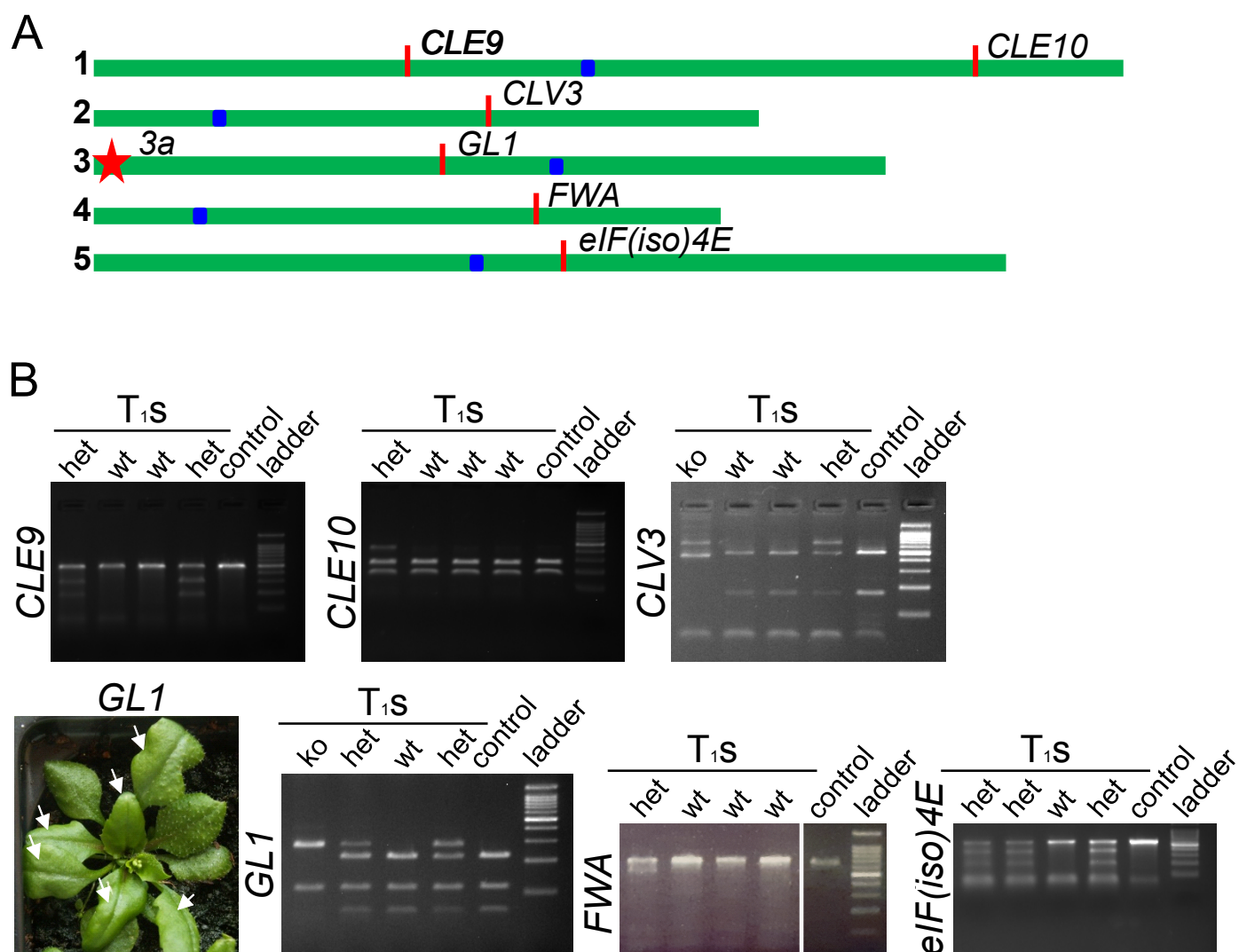

Supplemental Figure S3

Supplement: jkac105_Supplementary_Figure_S3 [file jkac105_supplementary_figure_s3.pdf]

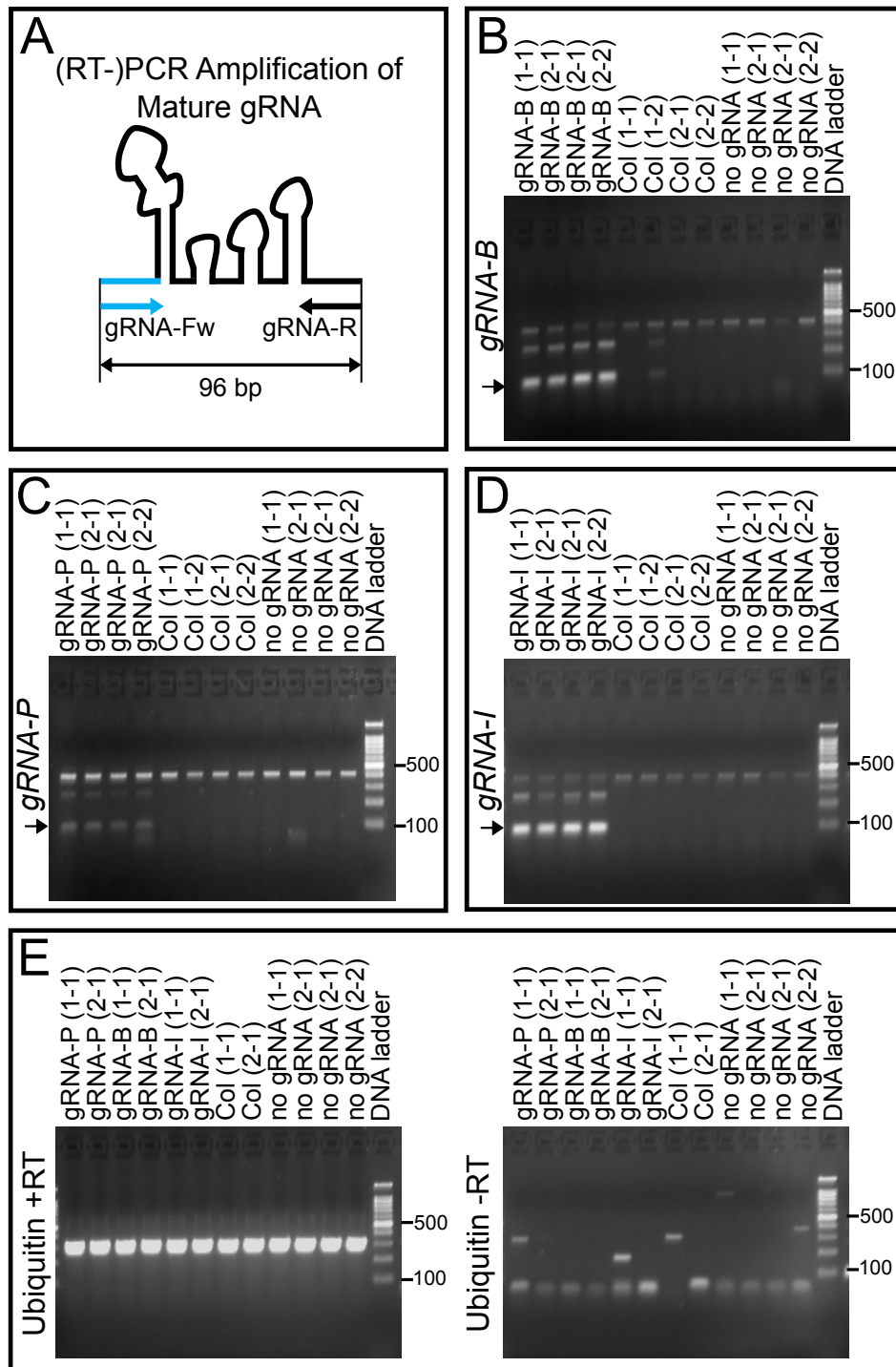

Supplemental Figure S4

Supplement: jkac105_Supplementary_Figure_S4 [file jkac105_supplementary_figure_s4.pdf]

Supplemental Figure S5.

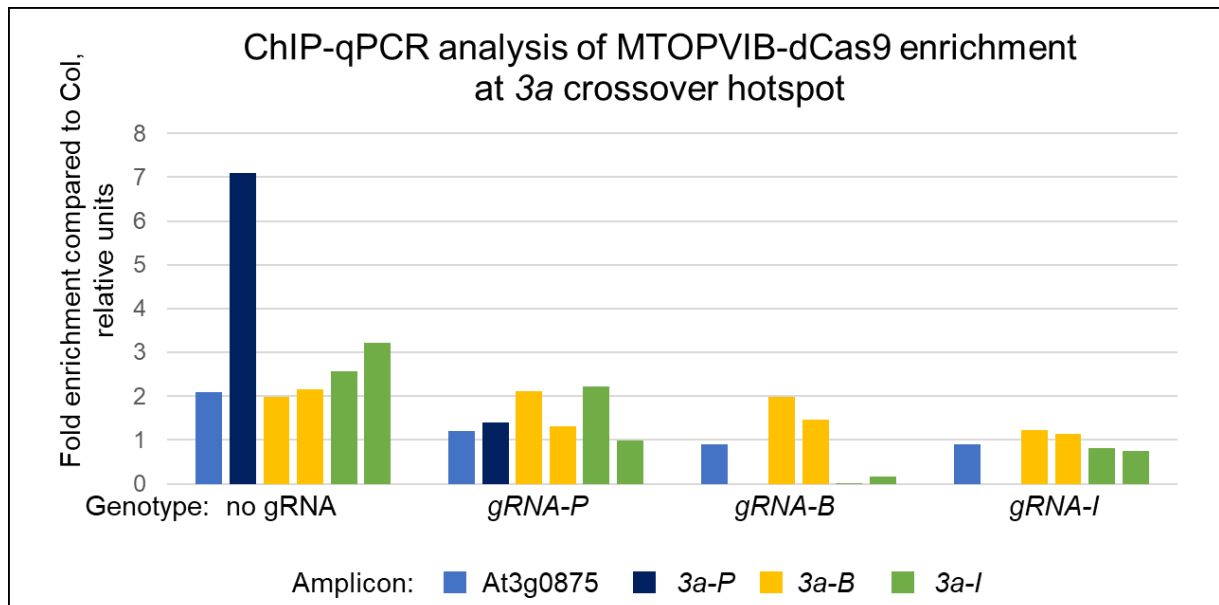

Supplement: jkac105_Supplementary_Figure_S5 [file jkac105_supplementary_figure_s5.pdf]
