# Supplementary material for: Coexpression of MEIOTIC-TOPOISOMERASE VIB-dCas9 with guide RNAs specific to a recombination hotspot is insufficient to increase crossover frequency in Arabidopsis
Source: G3 (Bethesda). 2022 Apr 29;12(7):jkac105. doi: 10.1093/g3journal/jkac105 (PMC9258527; doi:10.1093/g3journal/jkac105)
Supplement: jkac105_Supplementary_Tables [file jkac105_supplementary_tables.pdf]

SUPPLEMENTAL TABLES

Table 1. Oligonucleotides used in this study

Name	Sequence	Description
MTOPVI-Prom-SalI-F	ATATATAGTCGACTTTGCGCCAAAGGAAAATG	Cloning MTOPVIB-dCas9
MTOPVI-Term-NotI-R	ATATATATATATGCGGCCGCGATATCTTGCAGGGAAGTCAC	Cloning MTOPVIB-dCas9
MTOPVI-NheI-F	TCTTAGCTAGCTTAAGTATTTAAAGTTATATC	Cloning MTOPVIB-dCas9
MTOPVI-AscI-R	ATATACCATGGGGCGCGCCCAGAGATTCTCCGCTTTCG	Cloning MTOPVIB-dCas9
MTOPVIB-C-HA-top	GGAAGGCGGCAGCGGAGGCTCCCCTAAGAAAAAGCGCAAAGTCTATCCATATGACGTTCCAGATTACGCTTACCCTTACGATGTGCCTGACTACGCTTCTAGAGCTGGATCCTAAGTGCA	Cloning MTOPVIB-dCas9
MTOPVIB-C-HA-bottom	CTTAGGATCCAGCTCTAGAAGCGTAGTCAGGCACATCGTAAGGGTAAGCGTAATCTGGAACGTCATATGGATAGACTTTGCGCTTTTTCTTAGGGGAGCCTCCGCTGCCGCCTTCCTGCA	Cloning MTOPVIB-dCas9
dCas9-Xba-F	GGAAGATCGGTTCAACGCCTCCTCG	Cloning MTOPVIB-dCas9
dCas9-BamHI-R	ATATATAGGATCCTCACTTTTTCTTTTTTGCCTGGC	Cloning MTOPVIB-dCas9, genotyping *MTOPVIB-dCas9* transgene
dCas9-1stMut-F	GACAAGAAGTACAGCATCGGCCTGGCCATCGGCACCAACTCTGTGGGCTG	dCas9 mutagenesis
dCas9-1stMut-R	GCCCAGCCCACAGAGTTGGTGCCGATGGCCAGGCCGATGC	dCas9 mutagenesis
dCas9-2ndMut-R	CCGGCTGTCCGACTACGATGTGGACGCTATCGTGCCTCAG	dCas9 mutagenesis
dCas9-2ndMut-R	CAGAAAGCTCTGAGGCACGATAGCGTCCACATCGTAGTCG	dCas9 mutagenesis
MTOPVIB-seq3-F	GTGCTTGAGGATTTGGAAT	Genotyping *MTOPVIB-dCas9* transgene
MTOPVIB-end-F	TGTCTGGCGACTATGCTG	Genotyping *MTOPVIB-dCas9* transgene
MTOPVIB-stop-R	CTATTCCTGCAGCATAGTCG	Genotyping *MTOPVIB-dCas9* transgene
MTOP-genot-compl-F	AGAATGGTGCAAAGTTTAGG	Genotyping *mtopvib-2* complemented with *MTOPVIB-dCas9*
MTOP-genot-compl-R	AAATGACCTACTTTCCTGCTC	Genotyping *mtopvib-2* complemented with *MTOPVIB-dCas9*
DNA_template_02880_prom-R	TCTAGATCTGAAGTCACCGC	Target DNA amplification for *in vitro* and *in planta* gRNA testing

DNA_template_02880_body-F	GTCACGAGAAACCAACCACT	Target DNA amplification for *in vitro* and *in planta* gRNA testing
DNA_template_02880_body-R	GAGAAGTTGTCCCATCACG	Target DNA amplification for *in vitro* and *in planta* gRNA testing
DNA_template_interg-F	GCTACCTACATTCGCGTG	Target DNA amplification for *in vitro* and *in planta* gRNA testing
DNA_template_interg_R	TGTAGCTTCCGTTGTTCAG	Target DNA amplification for *in vitro* and *in planta* gRNA testing
In_vitro_R	AAAAGCACCGACTCGGTGCCAC	*in vitro* gRNA testing
gRNA-P1	GAAATTAATACGACTCACTATAGG GGTTTTTGGAAAATTTATTT GTTTTAGAGCTAGAAATAGC	*in vitro* gRNA testing
gRNA-P2	GAAATTAATACGACTCACTATAGG GTGTAAATTCTTCGTCTCCT GTTTTAGAGCTAGAAATAGC	*in vitro* gRNA testing
gRNA-P3	GAAATTAATACGACTCACTATAGG GGTCATAATTGCAGAGAAAA GTTTTAGAGCTAGAAATAGC	*in vitro* gRNA testing
gRNA-P4	GAAATTAATACGACTCACTATAGG ACAAAAGAAAGGAAAAACAG GTTTTAGAGCTAGAAATAGC	*in vitro* gRNA testing
gRNA-P5	GAAATTAATACGACTCACTATAGG ACTTATATCAGAAATAAAAA GTTTTAGAGCTAGAAATAGC	*in vitro* gRNA testing
gRNA-P6	GAAATTAATACGACTCACTATAGG GGCTAATCTTTGTTTGTCAA GTTTTAGAGCTAGAAATAGC	*in vitro* gRNA testing
gRNA-B1	GAAATTAATACGACTCACTATAGG GCTGTTCGTAACAGTGTCCG GTTTTAGAGCTAGAAATAGC	*in vitro* gRNA testing
gRNA-B2	GAAATTAATACGACTCACTATAGG GAACAGCGAGTAAAGCTCTC GTTTTAGAGCTAGAAATAGC	*in vitro* gRNA testing
gRNA-B3	GAAATTAATACGACTCACTATAGG TCTTTACCAATCGGTGGTAT GTTTTAGAGCTAGAAATAGC	*in vitro* gRNA testing
gRNA-B4	GAAATTAATACGACTCACTATAGG CCGGTTGGTTACTCTGTATT GTTTTAGAGCTAGAAATAGC	*in vitro* gRNA testing
gRNA-B5	GAAATTAATACGACTCACTATAGG GCACGGAGTCCACTGCGATG GTTTTAGAGCTAGAAATAGC	*in vitro* gRNA testing
gRNA-B6	GAAATTAATACGACTCACTATAGG GTTTCTTCTAATCAGTTAAA GTTTTAGAGCTAGAAATAGC	*in vitro* gRNA testing
gRNA-I1	GAAATTAATACGACTCACTATAGG GTAGAAAAAAGGCGGGAAGT	*in vitro* gRNA testing

	GTTTTAGAGCTAGAAATAGC	
gRNA-I2	GAAATTAATACGACTCACTATAGG GTTCCAACTCGTACCAGACT GTTTTAGAGCTAGAAATAGC	*in vitro* gRNA testing
gRNA-I3	GAAATTAATACGACTCACTATAGG GAAGAAAACATATTGTGATC GTTTTAGAGCTAGAAATAGC	*in vitro* gRNA testing
gRNA-I4	GAAATTAATACGACTCACTATAGG AACAAAGTCAACGACAAAAG GTTTTAGAGCTAGAAATAGC	*in vitro* gRNA testing
gRNA-I5	GAAATTAATACGACTCACTATAGG ACTATTTAGTTCAAGCTTAA GTTTTAGAGCTAGAAATAGC	*in vitro* gRNA testing
gRNA-I6	GAAATTAATACGACTCACTATAGG ATTTGACCTTAGCAAGTTGA GTTTTAGAGCTAGAAATAGC	*in vitro* gRNA testing
L5AD5-F-pChimera	CGGGTCTCAGGCAGGATGGCAGTCTGATTGAACAAAGCACCAGTGG	Cloning 6^{x}(pre-tRNA-gRNA) into pEnChimera or pChimera
L3AD5-R	TAGGTCTCCAAACGGATGAGCGACAGCAAACAAAAAAAAAAGCACCGACTCG	Cloning 6^{x}(pre-tRNA-gRNA) into pEnChimera or pChimera
S5AD5-F-pChimera	CGGGTCTCAGGCAGGATGGCAGTCTGATTG	Cloning 6^{x}(pre-tRNA-gRNA) into pEnChimera or pChimera
S3AD5-R	TAGGTCTCCAAACGGATGAGCGACAGCAAAC	Cloning 6^{x}(pre-tRNA-gRNA) into pEnChimera or pChimera
Sg-F-Pr6	TAGGTCTCCGAAAATTTATTTGTTTTAGAGCTAGAA	*gRNA-P* 6^{x}(pre-tRNA-gRNA) assembly
Sg-R-Pr6	ATGGTCTCATTTCCAAAAACCTGCACCAGCCGGGAA	*gRNA-P* 6^{x}(pre-tRNA-gRNA) assembly
Sg-F-Pr5	TAGGTCTCCTCTTCGTCTCCTGTTTTAGAGCTAGAA	*gRNA-P* 6^{x}(pre-tRNA-gRNA) assembly
Sg-R-Pr5	ATGGTCTCAAAGAATTTACACTGCACCAGCCGGGAA	*gRNA-P* 6^{x}(pre-tRNA-gRNA) assembly
Sg-F-Pr4	TAGGTCTCCTTGCAGAGAAAAGTTTTAGAGCTAGAA	*gRNA-P* 6^{x}(pre-tRNA-gRNA) assembly
Sg-R-Pr4	ATGGTCTCAGCAATTATGACCTGCACCAGCCGGGAA	*gRNA-P* 6^{x}(pre-tRNA-gRNA) assembly
Sg-F-Pr3	TAGGTCTCCAAGGAAAAACAGGTTTTAGAGCTAGAA	*gRNA-P* 6^{x}(pre-tRNA-gRNA) assembly
Sg-R-Pr3	ATGGTCTCACCTTTCTTTTGTTGCACCAGCCGGGAA	*gRNA-P* 6^{x}(pre-tRNA-gRNA) assembly
Sg-F-Pr1	TAGGTCTCCCAGAAATAAAAAGTTTTAGAGCTAGAA	*gRNA-P* 6^{x}(pre-tRNA-gRNA) assembly
Sg-R-Pr1	ATGGTCTCATCTGATATAAGTTGCACCAGCCGGGAA	*gRNA-P* 6^{x}(pre-tRNA-gRNA) assembly
Sg-F-Pr2	TAGGTCTCCTTTGTTTGTCAAGTTTTAGAGCTAGAA	*gRNA-P* 6^{x}(pre-tRNA-gRNA) assembly
Sg-r-Pr2	ATGGTCTCACAAAGATTAGCCTGCACCAGCCGGGAA	*gRNA-P* 6^{x}(pre-tRNA-gRNA) assembly
Sg-F-B1	TAGGTCTCCTAACAGTGTCCGGTTTTAGAGCTAGAA	*gRNA-B* 6^{x}(pre-tRNA-gRNA) assembly
Sg-R-B1	ATGGTCTCAGTTACGAACAGCTGCACCAGCCGGGAA	*gRNA-B* 6^{x}(pre-tRNA-gRNA) assembly
Sg-F-B2	TAGGTCTCCAGTAAAGCTCTCGTTTTAGAGCTAGAA	*gRNA-B* 6^{x}(pre-tRNA-gRNA) assembly

Sg-R-B2	ATGGTCTCA TACTCGCTGTTCTGCACCAGCCGGGAA	*gRNA-B* 6×(pre-tRNA-gRNA) assembly
Sg-F-B3	TAGGTCTCCAATCGGTGGTATGTTTTAGAGCTAGAA	*gRNA-B* 6×(pre-tRNA-gRNA) assembly
Sg-R-B3	ATGGTCTCAGATTGGTAAAGATGCACCAGCCGGGAA	*gRNA-B* 6×(pre-tRNA-gRNA) assembly
Sg-F-B4	TAGGTCTCCTTACTCTGTATTGTTTTAGAGCTAGAA	*gRNA-B* 6×(pre-tRNA-gRNA) assembly
Sg-R-B4	ATGGTCTCAGTAACCAACCGGTGCACCAGCCGGGAA	*gRNA-B* 6×(pre-tRNA-gRNA) assembly
Sg-F-B5	TAGGTCTCCTCCACTGCGATGGTTTTAGAGCTAGAA	*gRNA-B* 6×(pre-tRNA-gRNA) assembly
Sg-R-B5	ATGGTCTCATGGACTCCGTGCTGCACCAGCCGGGAA	*gRNA-B* 6×(pre-tRNA-gRNA) assembly
Sg-F-B6	TAGGTCTCCTAATCAGTTAAAGTTTTAGAGCTAGAA	*gRNA-B* 6×(pre-tRNA-gRNA) assembly
Sg-R-B6	ATGGTCTCAATTAGAAGAAACTGCACCAGCCGGGAA	*gRNA-B* 6×(pre-tRNA-gRNA) assembly
Sg-F-Int1	TAGGTCTCCAAGGCGGGAAGTGTTTTAGAGCTAGAA	*gRNA-I* 6×(pre-tRNA-gRNA) assembly
Sg-R-Int1	ATGGTCTCACCTTTTTTCTACTGCACCAGCCGGGAA	*gRNA-I* 6×(pre-tRNA-gRNA) assembly
Sg-F-Int2	TAGGTCTCCTCGTACCAGACTGTTTTAGAGCTAGAA	*gRNA-I* 6×(pre-tRNA-gRNA) assembly
Sg-R-Int2	ATGGTCTCAACGAGTTGGAACTGCACCAGCCGGGAA	*gRNA-I* 6×(pre-tRNA-gRNA) assembly
Sg-F-Int3	TAGGTCTCCCATATTGTGATCGTTTTAGAGCTAGAA	*gRNA-I* 6×(pre-tRNA-gRNA) assembly
Sg-R-Int3	ATGGTCTCATATGTTTTCTTCTGCACCAGCCGGGAA	*gRNA-I* 6×(pre-tRNA-gRNA) assembly
Sg-F-Int4	TAGGTCTCCCAACGACAAAAGGTTTTAGAGCTAGAA	*gRNA-I* 6×(pre-tRNA-gRNA) assembly
Sg-R-Int4	ATGGTCTCAGTTGACTTTGTTTGCACCAGCCGGGAA	*gRNA-I* 6×(pre-tRNA-gRNA) assembly
Sg-F-Int5	TAGGTCTCCGTTCAAGCTTAAGTTTTAGAGCTAGAA	*gRNA-I* 6×(pre-tRNA-gRNA) assembly
Sg-R-Int5	ATGGTCTCAGAACTAAATAGTTGCACCAGCCGGGAA	*gRNA-I* 6×(pre-tRNA-gRNA) assembly
Sg-F-Int6	TAGGTCTCCTTAGCAAGTTGAGTTTTAGAGCTAGAA	*gRNA-I* 6×(pre-tRNA-gRNA) assembly
Sg-R-Int6	ATGGTCTCACTAAGGTCAAATTGCACCAGCCGGGAA	*gRNA-I* 6×(pre-tRNA-gRNA) assembly
DNA_template_02880_body-F	TGTCTTCTATCTCGCTGCG	*in planta gRNA* testing
DNA_template_02880_body-R	ACAGGAGCTTCAACATTCC	*in planta gRNA* testing
3a-int-templ-fw-v3	TGGGGTCAAGTTGCAATTTCT	*in planta gRNA* testing
3a-int-templ-rev-v3	GTTCTATTTGCTTCCACGGTTTA	*in planta gRNA* testing
eIF(iso)4E _Fw	AAGAGTTAAATGCTCTGATGGAC	*in planta gRNA* testing
eIF(iso)4E _Rv	ACAAGTGAATTTCAGCATTCG	*in planta gRNA* testing
CLE10-fw	CGTCTCAATTGATGTAGCAGCA	*in planta gRNA* testing
CLE10-rev	GACGCCGTACCTTTGATCAA	*in planta gRNA* testing
CLE9-fw	TGTGCTTCTTCTGTGTTGCA	*in planta gRNA* testing
CLE9-rev	GGTTTTAGTTGTGAAGCGGGT	*in planta gRNA* testing
GLBR-fw	TCAACTTAACCGGCCAAATCT	*in planta gRNA* testing
GLRB-rev	GCACTGGCCAATGGAACC	*in planta gRNA* testing
FWA-fw	AGCGTCTACCAAATCTACACTTT	*in planta gRNA* testing

FWA-rev	GCGCTCGTATGAATGTTGAATG	*in planta gRNA* testing
CLV3-F A00954	TCTCGCCCTTGTAGGCTTACG	*in planta gRNA* testing
CLV3-R A02289	GCTGAAAGTTGTTTCTTGGCTG	*in planta gRNA* testing
Pr-RT-F1	ACTTATATCAGAAATAAAAA	RT-PCR
Pr-RT-F2	GGCTAATCTTTGTTTGTCAA	RT-PCR
Pr-RT-F3	ACAAAAGAAAGGAAAAACAG	RT-PCR
Pr-RT-F4	GGTCATAATTGCAGAGAAAA	RT-PCR
Pr-RT-F5	GTGTAAATTCTTCGTCTCCT	RT-PCR
Pr-RT-F6	GGTTTTTGGAAAATTTATTT	RT-PCR
AG-RT-F1	GCTGTTCGTAACAGTGTCCG	RT-PCR
AG-RT-F2	GAACAGCGAGTAAAGCTCTC	RT-PCR
AG-RT-F3	TCTTTACCAATCGGTGGTAT	RT-PCR
AG-RT-F5	GCACGGAGTCCACTGCGATG	RT-PCR
AG-RT-F6	GTTTCTTCTAATCAGTTAAA	RT-PCR
Interg-RT-F1	GTAGAAAAAAGGCGGGAAGT	RT-PCR
Interg-RT-F2	GTTCCAACTCGTACCAGACT	RT-PCR
Interg-RT-F3	GAAGAAAACATATTGTGATC	RT-PCR
Interg-RT-F4	AACAAAGTCAACGACAAAAG	RT-PCR
Interg-RT-F5	ACTATTTAGTTCAAGCTTAA	RT-PCR
Interg-RT-F6	ATTTGACCTTAGCAAGTTGA	RT-PCR
Ub-RT-F	CAGATCGATAGCAGCACCTTGG	RT-PCR
Ub-RT-R	GGAAATGGCATCGAAACGG	RT-PCR
gRNA-R	GCACCGACTCGGTGCCAC	RT-PCR
SNP1v3-KASP-Rev-A	GAAGGTGACCAAGTTCATGCTCCATTGGACTTACGATTGGA	KASP assay
SNP1v3-KASP-Rev-B	GAAGGTCGGAGTCAACGGATTCCATTGGACTTACGATTGGG	KASP assay
SNP1v3-KASP-Fw	AACTTGAAACAATGGAATACACAATC	KASP assay
SNP1-KASP-Fw-A	GAAGGTGACCAAGTTCATGCTTTTCTCTCGTTCCAAGACAGCG	KASP assay
SNP1-KASP-Fw-B	GAAGGTCGGAGTCAACGGATTTTTCTCTCGTTCCAAGACAGCA	KASP assay
SNP1-KASP-Rev	TTCAAAGTCCATGTACGTTGG	KASP assay
SNP2v3-KASP-Fw-A	GAAGGTGACCAAGTTCATGCTCCCAATGGCCTATATGTCAA	KASP assay
SNP2v3-KASP-Fw-B	GAAGGTCGGAGTCAACGGATTCCCAATGGCCTATATGTCAT	KASP assay
SNP2v3-KASP-Rev	ATCACATGTAGGCCTTTAAGGTTC	KASP assay
SNP3-KASP-Rev-A	GAAGGTGACCAAGTTCATGCTACCCTCATAAGATTCACCAAACT	KASP assay
SNP3-KASP-Rev-B	GAAGGTCGGAGTCAACGGATTACCCTCATAAGATTCACCAAACC	KASP assay

SNP3-KASP-Fw	TGTCTTTGATTGCTAATTGTTATGTG	KASP assay
SNP4-KASP-Fw-A	GAAGGTGACCAAGTTCATGCTGATTTTCGTGCTGGGGG	KASP assay
SNP4-KASP-Fw-B	GAAGGTCGGAGTCAACGGATTGATTTTCGTGCTGGGGA	KASP assay
SNP4-KASP-Rev	TCAACAAAGAAGTAAGGCGAG	KASP assay
SNP5-KASP-Fw-A	GAAGGTGACCAAGTTCATGCTTCGCTGTTCGTAACAGTGTC	KASP assay
SNP5-KASP-Fw-B	GAAGGTCGGAGTCAACGGATTTCGCTGTTCGTAACAGTGTT	KASP assay
SNP5-KASP-Rev	TGGACTCCGTGCCAATTA	KASP assay
SNP5v3-KASP-Fw-A	GAAGGTGACCAAGTTCATGCTCCTCGAACTGCTTTTGAAGGT	KASP assay
SNP5v3-KASP-Fw-B	GAAGGTCGGAGTCAACGGATTCCTCGAACTGCTTTTGAAGGC	KASP assay
SNP5v3-KASP-Rev	CTCTCTGCCTCACAAGTGTCT	KASP assay
SNP5v4-KASP-Rev-A	GAAGGTGACCAAGTTCATGCTGGCGTATTGGGTCCTCCA	KASP assay
SNP5v4-KASP-Rev-B	GAAGGTCGGAGTCAACGGATTGGCGTATTGGGTCCTCCT	KASP assay
SNP5v4-KASP-Fw	ACACTCTCTGTGGGAAGCC	KASP assay
SNP6-KASP-Fw-A	GAAGGTGACCAAGTTCATGCTGCGATATTGCACGGTATAGAATA	KASP assay
SNP6-KASP-Fw-B	GAAGGTCGGAGTCAACGGATTGCGATATTGCACGGTATAGAATC	KASP assay
SNP6-KASP-Rev	CAAGATCACAAACTAGCATCAGC	KASP assay
SNP7-KASP-Rev-A	GAAGGTGACCAAGTTCATGCTGGATTCTGGGATAAGATGTGTAAA	KASP assay
SNP7-KASP-Rev-B	GAAGGTCGGAGTCAACGGATTGGATTCTGGGATAAGATGTGTAAT	KASP assay
SNP7-KASP-Fw	AAAATGTCTATAAAACTATTGCTTTTGC	KASP assay
SNP6v2-KASP-Rev-A	GAAGGTGACCAAGTTCATGCTAGTCTGGTACGAGTTGGAACAT	KASP assay
SNP6v2-KASP-Rev-B	GAAGGTCGGAGTCAACGGATTAGTCTGGTACGAGTTGGAACAC	KASP assay
SNP6v2-KASP-Fw	TCATGTGATATACCATTTACACCG	KASP assay
SNP6v3-KASP-Rev-A	GAAGGTGACCAAGTTCATGCTTGTGTCATGGCCGAACTTAT	KASP assay
SNP6v3-KASP-Rev-B	GAAGGTCGGAGTCAACGGATTTGTGTCATGGCCGAACTTAA	KASP assay
SNP6v3-KASP-Fw	ATTTTTGTAGGCTCAAATCAATTG	KASP assay
SNP8-KASP-Fw	CCAGGAGGAACACAAAGACA	KASP assay
SNP8-KASP-Rev-A	GAAGGTGACCAAGTTCATGCTGAGTGCAACTCAAAGTGTAGCTT	KASP assay
SNP8-KASP-Rev-B	GAAGGTCGGAGTCAACGGATTGAGTGCAACTCAAAGTGTAGCTA	KASP assay
SNP8v2-KASP-Fw-A	GAAGGTGACCAAGTTCATGCTACTTCAATATCAGAAAACTAATAGC CA	KASP assay
SNP8v2-KASP-Fw-B	GAAGGTCGGAGTCAACGGATTACTTCAATATCAGAAAACTAATAGC CG	KASP assay
SNP8v2-KASP-Rev	CTCGTGGAGGTAAAAATGACC	KASP assay
SNP8v3-KASP-Rev-A	GAAGGTGACCAAGTTCATGCTATTTTCCAAACAGAGCGTTTAGAC	KASP assay

SNP8v3-KASP-Rev-B	GAAGGTCGGAGTCAACGGATTATTTTCCAAACAGAGCGTTTAGAA	KASP assay
SNP8v3-KASP-Fw	AACATTTCTCTCAGTGAAAATTGG	KASP assay
SNP8v4-KASP-Fw-A	GAAGGTGACCAAGTTCATGCTTGGAAAATTATATTGTGTAGTTGACTAGTG	KASP assay
SNP8v4-KASP-Fw-B	GAAGGTCGGAGTCAACGGATTTGGAAAATTATATTGTGTAGTTGACTAGTT	KASP assay
SNP8v4-KASP-Rev	ACTCGCTTATTTTCCCCTTTC	KASP assay
SNP9v3-KASP-Rev-A	GAAGGTGACCAAGTTCATGCTAAATATTTAAGTTTTCTTCTTAAGCCT	KASP assay
SNP9v3-KASP-Rev-B	GAAGGTCGGAGTCAACGGATTAAATATTTAAGTTTTCTTCTTAAGCCA	KASP assay
SNP9v3-KASP-Fw	TTAAAATTAACTTCCAATGAAATTACC	KASP assay
SNP10-KASP-Fw-A	GAAGGTGACCAAGTTCATGCTTTTTCCGGTTCAAAGTCAGAT	KASP assay
SNP10-KASP-Fw-B	GAAGGTCGGAGTCAACGGATTTTTTCCGGTTCAAAGTCAGAC	KASP assay
SNP10-KASP-Rev	CAGAAGAACGCAAAAGATTGTC	KASP assay
SNP11v2-KASP-Fw-A	GAAGGTGACCAAGTTCATGCTCTCATTACTCATCTTTCAATAGCCACT	KASP assay
SNP11v2-KASP-Fw-B	GAAGGTCGGAGTCAACGGATTCTCATTACTCATCTTTCAATAGCCACG	KASP assay
SNP11v2-KASP-Rev	TACCTTTAGGGGACTGGAGCT	KASP assay
SNP11v3-KASP-Fw-A	GAAGGTGACCAAGTTCATGCTTTGAAGTCCAATTCGTTTAATTCTTT	KASP assay
SNP11v3-KASP-Fw-B	GAAGGTCGGAGTCAACGGATTTTGAAGTCCAATTCGTTTAATTCTTC	KASP assay
SNP11v3-KASP-Rev	GGAAGCATATCATCATCCACTG	KASP assay
SNP11v4-KASP-Fw-A	GAAGGTGACCAAGTTCATGCTCACGACAGAAGACTGGGAAT	KASP assay
SNP11v4-KASP-Fw-B	GAAGGTCGGAGTCAACGGATTCACGACAGAAGACTGGGAAC	KASP assay
SNP11v4-KASP-Rev	CATCTACTTGACATTGATTTGCCTA	KASP assay
SNP12-KASP-Fw-A	GAAGGTGACCAAGTTCATGCTGTATATAATTGTGTTTTGTTTCACATGC	KASP assay
SNP12-KASP-Fw-B	GAAGGTCGGAGTCAACGGATTGTATATAATTGTGTTTTGTTTCACATGG	KASP assay
SNP12-KASP-Rev	CATTTAATATATTTCGTCAGTGGATTAGG	KASP assay
at3g02875_ex1_F2	ACCTTCTTCTTTCTCCCTCCTC	ChIP-qPCR
at3g02875_ex1_R2	ACTCAGGATCCTTGGCTGAA	ChIP-qPCR

Pr-Ch_q_F1	CCTTTCAAATTGATACAACAA	ChIP-qPCR
Pr-Ch_q_R1	TGCCAGGTCTGTCAAATG	ChIP-qPCR
Pr-Ch_q_F2	GTTCAAACACTCGTTTACGC	ChIP-qPCR
Pr-Ch_q_R2	CTTCATCTTCTTCTTCTTCTTC	ChIP-qPCR
B_Ch_q_F1	TAGAGTCTGACCGGAGAGCT	ChIP-qPCR
B_Ch_q_R1	TGGACTCCGTGCCAATTACA	ChIP-qPCR
B_Ch_q_F3	CCTGAGATCACGCTTCCTCT	ChIP-qPCR
B_Ch_q_R3	TTCAAAAGCAGTTCGAGGCC	ChIP-qPCR
3a_int_ChiPq_F1	GTTCAAGCTTAAAGGGAAATCGA	ChIP-qPCR
3a_int_ChiPq_R1	TGCCCATGACTCGGTGTAAA	ChIP-qPCR
3a_int_ChiPq_F2	TGTGATATACCATTTACACCGAG	ChIP-qPCR
3a_int_ChiPq_R2	TTTATTGAACCCCATGTGCA	ChIP-qPCR

Table 2. Sequences and coordinates of guide RNAs used in this study

Guide RNA sequence	Target site	Genome coordinates	Target locus
GGTTTTTGGAAAATTTATTT	AT3G02880 promoter and 5' end	Chr3: 634,713-634,732	*3a-P*
GTGTAAATTCTTCGTCTCCT	AT3G02880 promoter and 5' end	Chr3: 634,776-634,795	*3a-P*
GGTCATAATTGCAGAGAAAA	AT3G02880 promoter and 5' end	Chr3: 634,582-634,601	*3a-P*
ACAAAAGAAAGGAAAAACAG	AT3G02880 promoter and 5' end	Chr3: 634,330-634,349	*3a-P*
ACTTATATCAGAAATAAAAA	AT3G02880 promoter and 5' end	Chr3: 634,146-634,165	*3a-P*
GGCTAATCTTTGTTTGTCAA	AT3G02880 promoter and 5' end	Chr3: 643,178-643,197	*3a-P*
GCTGTTCGTAACAGTGTCCG	AT3G02880 gene body	Chr3: 634,921-634,940	*3a-B*
GAACAGCGAGTAAAGCTCTC	AT3G02880 gene body	Chr3: 634,908-634,927	*3a-B*
TCTTTACCAATCGGTGGTAT	AT3G02880 gene body	Chr3: 635,056-635,075	*3a-B*

CCGGTTGGTTACTCTGTATT	AT3G02880 gene body	Chr3: 635,304-635,323	*3a-B*
GCACGGAGTCCACTGCGATG	AT3G02880 gene body	Chr3: 634,989-635,008	*3a-B*
GTTTCTTCTAATCAGTTAAA	AT3G02880 gene body	Chr3: 635,386-635,405	*3a-B*
GTAGAAAAAAGGCGGGAAGT	Intergenic between AT3G02880 and AT3G02885	Chr3: 637,059-637,078	*3a-I*
GTTCCAACTCGTACCAGACT	Intergenic between AT3G02880 and AT3G02885	Chr3: 637,756-637,775	*3a-I*
GAAGAAAACATATTGTGATC	Intergenic between AT3G02880 and AT3G02885	Chr3: 637,665-637,684	*3a-I*
AACAAAGTCAACGACAAAAG	Intergenic between AT3G02880 and AT3G02885	Chr3: 637,380-637,399	*3a-I*
ACTATTTAGTTCAAGCTTAA	Intergenic between AT3G02880 and AT3G02885	Chr3: 637,611-637,630	*3a-I*
ATTTGACCTTAGCAAGTTGA	Intergenic between AT3G02880 and AT3G02885	Chr3: 637,921-637,940	*3a-I*
AACTCACCGAACCGTCCCTA	AT1G69320, *CLE10*	Chr1: 26,062,017-26,062,036	non-3a gRNAs
ACGAACGGTGAGGAGTCACG	AT1G26600, *CLE9*	Chr1: 9,191,857-9,191,876	non-3a gRNAs
TGAGACCAGAAGCATCATGA	AT2G27250, *CLV3*	Chr2: 11,665,672-11,665,691	non-3a gRNAs
GGAAAAGTTGTAGACTGAGA	AT3G27920, *GL1*	Chr3: 10,363,224-10,363, 243	non-3a gRNAs
ACGGAAAGATGTATGGGCTT	AT4G25530, *FWA*	Chr4: 13,038,251-13,038,270 and	non-3a gRNAs

		Chr4: 13,038,296-13,038,315	
TGTGAACGAGCCTCTCCCGG	AT5G35620, *eIF(iso)4A*	Chr5: 13,826,298-13,826,317	non-3a gRNAs

Table 3. Seed sets in Col, *mtopvib* and complementing *MTOPVIB-dCas9 mtopvib* lines. Two-tailed t-test was used to calculate the *P* values

Genotype	Total siliques scored	Total seeds scored	Average	Standard Deviation	*P value*
**Col**	10	645	64.5	8.2	
mtopvib	56	105	1.9	1.7	<0.00001
MTOPVIB-dCas9 mtopvib	7	463	66.1	3.3	0.63

Table 4. Genetic distances of *CTL2.10* in wild type and *MTOPVIB-dCas9 mtopvib*. cM were calculated using the formula: cM = 100 × (1 – $[1\text{-}2(N_G+N_R)/N_T]^{½}$), where N_G is a number of green-alone fluorescent seeds, N_R is a number of red-alone fluorescent seed and N_T is the total number of seeds counted.

Genotype	Green-alone	Red-alone	Both red and green	Neither red or green	Total	CTL 2.10, cM
Wild type	140	152	1744	467	2503	12.44
Wild type	133	129	1611	453	2326	11.98
Wild type	131	147	1649	447	2374	12.49
Wild type	131	166	1727	440	2464	12.88
Wild type	134	161	1734	477	2506	12.56
Wild type	145	163	1621	437	2366	14.00

Wild type	109	169	1695	449	2422	12.23
Wild type	136	139	1648	445	2368	12.38
MTOPVIB-dCas9 mtopvib	120	153	1665	449	2387	12.18
MTOPVIB-dCas9 mtopvib	138	174	1678	423	2413	13.90
MTOPVIB-dCas9 mtopvib	124	143	1597	413	2277	12.51
MTOPVIB-dCas9 mtopvib	131	146	1588	416	2281	12.99
MTOPVIB-dCas9 mtopvib	127	157	1681	463	2428	12.47
MTOPVIB-dCas9 mtopvib	145	169	1675	405	2394	14.11
MTOPVIB-dCas9 mtopvib	148	159	1648	431	2386	13.82
MTOPVIB-dCas9 mtopvib	147	174	1656	425	2402	14.40
MTOPVIB-dCas9 mtopvib	112	143	1627	417	2299	11.79
MTOPVIB-dCas9 mtopvib	133	164	1656	493	2446	12.99
MTOPVIB-dCas9 mtopvib	127	141	1599	433	2300	12.42

Table 5. Genetic distances of *CTL5.1* in wild type and *MTOPVIB-dCas9 mtopvib*. cM were calculated using the formula: cM = 100 × (1 – [1-2(N_G+N_R)/N_T]^{½}), where N_G is a number of green-alone fluorescent seeds, N_R is a number of red-alone fluorescent seed and N_T is the total number of seeds counted.

Genotype	Green-alone	Red-alone	Both red and green	Neither red or green	Total	CTL 5.1, cM

Wild type	220	233	1455	353	2261	22.59
Wild type	276	264	1477	364	2381	26.08
Wild type	242	246	1532	332	2352	23.51
Wild type	264	275	1586	356	2481	24.80
Wild type	250	251	1661	379	2541	22.18
Wild type	280	237	1633	343	2493	23.50
Wild type	239	257	1648	355	2499	22.34
Wild type	193	225	1217	254	1889	25.34
Wild type	264	241	1559	363	2427	23.59
MTOPVIB-dCas9 mtopvib	229	234	1479	334	2276	22.98
MTOPVIB-dCas9 mtopvib	271	248	1562	306	2387	24.82
MTOPVIB-dCas9 mtopvib	273	272	1544	349	2438	25.64
MTOPVIB-dCas9 mtopvib	251	204	1354	339	2148	24.08
MTOPVIB-dCas9 mtopvib	269	247	1551	376	2443	24.00
MTOPVIB-dCas9 mtopvib	249	212	1489	327	2277	22.86
MTOPVIB-dCas9 mtopvib	259	241	1515	382	2397	23.66
MTOPVIB-dCas9 mtopvib	280	251	1621	360	2512	24.02
MTOPVIB-dCas9 mtopvib	286	228	1482	343	2339	25.13

MTOPVIB-dCas9 mtopvib	288	250	1542	364	2444	25.18
MTOPVIB-dCas9 mtopvib	250	209	1555	345	2359	21.84
MTOPVIB-dCas9 mtopvib	319	240	1566	388	2513	25.49
MTOPVIB-dCas9 mtopvib	254	221	1639	358	2472	21.53
MTOPVIB-dCas9 mtopvib	230	219	1457	334	2240	22.60

Table 6. Coordinates of *3a* crossover hotspot and genes within *3a*

Locus	Chromosome	Start coordinate	End coordinate
3a crossover hotspot	3	634,109	639,934
AT3G02880	3	634,465	637,284
AT3G02885	3	637,921	639,178

Table 7. CRISPR/Cas9 gene editing efficiencies in *3a*. Number and percentage of *Cas9-gRNA-P*, *Cas9-gRNA-B* and *Cas9-gRNA-I* T_1 progeny with gene editing events and gene editing efficiencies for individual guide RNAs.

Transgenic line (grey) or guide RNA	Total number of T_1s analysed	Number of T_1s with mutation	% T_1s with gene editing
Cas9-gRNA-P	90	8	8.9
gRNA-P-1	90	3	3.3
gRNA-P-2	90	0	0
gRNA-P-3	90	2	2.2
gRNA-P-4	90	4	4.4
gRNA-P-5	90	2	2.2
gRNA-P-6	90	4	4.4
Cas9-gRNA-B	33	4	12.1
gRNA-B-1	33	3	9.1

gRNA-B-2	33	2	6.1
gRNA-B-3	33	1	3.0
gRNA-B-4	33	1	3.0
gRNA-B-5	33	2	6.1
gRNA-B-6	33	2	6.1
Cas9-gRNA-I	66	17	25.8
gRNA-I-1	66	4	6.1
gRNA-I-2	66	13	19.7
gRNA-I-3	66	1	1.5
gRNA-I-4	66	4	6.1
gRNA-I-5	66	14	21.2
gRNA-I-6	66	15	22.8

Table 8. CRISPR/Cas9 gene editing efficiencies outside of *3a* hotspot.

**Guide RNA target site**	**Wild type**	**One allele affected**	**Both alleles affected**	**% T_1s with mutation**
CLE9	46	2	0	4.2
CLE10	45	3	0	6.3
CLV3	32	14	2	33.3
GL1	22	24	2	50.0
FWA	47	1	0	2.1
eIF(iso)4E	30	18	0	37.5

Table 9. *3a* crossover frequencies in Col/Ws *MTOPVIB-dCas9 mtopvib* F_1 populations in the presence or absence of guide RNAs targeting *3a*. Chi-square test was used to calculate *P*-values.

Genotype	Crossovers per microliter	Parentals per microliter	*3a*	Standard Deviation	*3a*	*P*-value
non-3a gRNA	26.9	17412	0.155 cM	0.014	26.4 cM/Mb	

no gRNA	32.5	24767	0.131 cM	0.011 cM	22.3 cM/Mb	0.56
gRNA-P	45.6	30059	0.152 cM	0.014 cM	25.9 cM/Mb	0.96
gRNA-B	42.3	22375	0.189 cM	0.021 cM	32.2 cM/Mb	0.44
gRNA-I	41.2	23522	0.175 cM	0.020 cM	29.8 cM/Mb	0.64

Table 10. Fine-scale mapping of *3a* crossover frequencies in Col/Ws *MTOPVIB-dCas9 mtopvib* F$_1$ populations in the presence or absence of guide RNAs targeting *3a*.

Coordinate	Interval Length	CO 'no gRNA'	cM/Mb 'no gRNA'	CO 'non-3a gRNA'	cM/Mb 'non-3a gRNA'	CO *gRNA-P*	cM/Mb *gRNA-P*	CO *gRNA-B*	cM/Mb *gRNA-B*	CO *gRNA-I*	cM/Mb *gRNA-I*
634109											
634938	829	0	0.0	0	0.0	0	0.0	0	0.0	0	0.0
635457	519	5	15.8	2	7.8	0	0.0	2	8.4	2	7.7
635520	63	0	0.0	1	32.0	0	0.0	1	34.5	0	0.0
636119	599	3	8.2	3	10.1	2	5.6	3	10.9	0	0.0
637283	1164	6	8.4	9	15.6	13	18.9	14	26.1	13	22.2
637754	471	8	27.8	6	25.6	8	28.7	11	50.7	13	54.9
637900	146	3	33.6	0	0.0	0	0.0	0	0.0	3	40.9
637952	52	0	0.0	0	0.0	0	0.0	0	0.0	0	0.0
637974	22	0	0.0	0	0.0	0	0.0	0	0.0	0	0.0
637988	14	0	0.0	0	0.0	0	0.0	0	0.0	0	0.0
638139	151	4	43.4	3	40.0	6	67.1	3	43.2	2	26.3
638196	57	3	86.2	1	35.3	1	29.6	0	0.0	1	34.9
638483	287	7	39.9	8	56.1	17	100.0	12	90.8	20	138.6
638786	303	14	75.7	13	86.4	20	111.5	10	71.7	12	78.8
638818	32	1	51.2	0	0.0	1	52.8	1	67.9	1	62.1
639370	552	13	38.6	25	91.2	16	49.0	20	78.7	12	43.2
639538	168	13	126.7	6	71.9	6	60.3	10	129.3	9	106.5
639934	462	0	0.0	0	0.0	0	0.0	0	0.0	0	0.0
Total	5825	80	22.49	77	26.61	90	26.09	87	32.45	88	30.04

Table 11. Genomic positions of single nucleotide polymorphisms (SNPs), in red, used for KASP assays. Assays highlighted in grey were used for initial screening of the F_2 population. Assays in white were used only on individual F_2 plants to determine gene conversion tract lengths.

KASP assay	Position in the genome	Distance between SNPs, bp	Sequence in Col	Sequence in Ws
SNP1v3	628865		ACTCCAACAACAGGGACTTTT	ACTCCAACAACAGGGACTTTC
SNP1	630828	1963	CCTTTCTCTCGTTCCAAGACAGCG	CCTTTCTCTCGTTCCAAGACAGCA
SNP2v3	631531	703	AAGAACCCAATGGCCTATATGTCAA	AAGAACCCAATGGCCTATATGTCAT
SNP3	632767	1236	TTGTTAGTGTGTGTGTCTTATGAGA	TTGTTAGTGTGTGTGTCTTATGAGG
SNP4	633695	928	AAGGCTGCGATTTTCGTGCTGGGGG	AAGGCTGCGATTTTCGTGCTGGGGA
SNP5	634938	1243	GCTGTTCGTAACAGTGTC	GCTGTTCGTAACAGTGTT
SNP5v3	635457	519	CGAACTGCTTTTGAAGGT	CGAACTGCTTTTGAAGGC
SNP5v4	635520	63	CCCAATGGTGGTGACGCT	CCCAATGGTGGTGACGCA
SNP6	636119	599	ATTGCACGGTATAGAATA	ATTGCACGGTATAGAATC
SNP7	637283	1164	AATAAATTGGTTTTTGTTTTCTTCT	AATAAATTGGTTTTTGTTTTCTTCA
SNP6v2	637754	471	AGTTGCCACAACTTTATCTCA	AGTTGCCACAACTTTATCTCG
SNP6v3	638139	385	TTTCATAAATATGTAACCATAA	TTTCATAAATATGTAACCATAT
SNP8	638483	344	GTGATGTTGCTGAACAACGGA	GTGATGTTGCTGAACAACGGT
SNP8v2	638818	335	TCAATATCAGAAAACTAATAGCCA	TCAATATCAGAAAACTAATAGCCG
SNP8v3/8v4	639370	552	ATTATATTGTGTAGTTGACTAGTG	ATTATATTGTGTAGTTGACTAGTT
SNP9v3	640000	1517	TAATAATGTAATTCTAAATTA	TAATAATGTAATTCTAAATTT
SNP10	641219	1219	TTTTCCGGTTCAAAGTCAGAT	TTTTCCGGTTCAAAGTCAGAC
SNP11v2	642469	1250	TACTCATCTTTCAATAGCCACT	TACTCATCTTTCAATAGCCACG
SNP11v3	642671	202	AAGTCCAATTCGTTTAATTCTTT	AAGTCCAATTCGTTTAATTCTTC
SNP11v4	642735	64	GACAGAAGACTGGGAAT	GACAGAAGACTGGGAAC
SNP12	644083	1348	TAATTGTGTTTTGTTTCACATGC	TAATTGTGTTTTGTTTCACATGG

Table 12. Gene conversion events observed in F_2 progenies of Col/Ws *MTOPVIB-dCas9 mtopvib* in the presence or absence of guide RNAs targeting *3a*.

Genotype	Total F_2s analysed	Non-crossovers observed	Non-crossover frequency (%)
no gRNA	473	0	0
gRNA-P	469	2	0.43
gRNA-B	96	0	0
gRNA-I	96	0	0

Table 13. Gene conversion events observed in two individuals (#519 and #343) of F_2 progeny of Col/Ws *MTOPVIB-dCas9 gRNA-P mtopvib*.

Polymorphism Coordinate	Interval Length	Genotype	
		***gRNA-P*, F_2#519**	***gRNA-P*, F_2#343**
634109		Het	Ws
634938	829	Het	Ws
635457	519	Het	Ws
635520	63	Het	Ws
636119	599	Het	Het
637283	1164	Het	Ws
637754	471	Ws	Not tested
637900	146	Ws	Not tested
637952	52	Ws	Not tested
637974	22	Ws	Not tested
637988	14	Ws	Not tested
638139	151	Ws	Not tested
638196	57	Ws	Not tested
638483	287	Ws	Ws
638786	303	Het	Not tested
638818	32	Not tested	Not tested
639370	552	Not tested	Not tested

639538	168	Not tested	Not tested
640000	462	Het	Ws
