# Supplementary material for: Coexpression of MEIOTIC-TOPOISOMERASE VIB-dCas9 with guide RNAs specific to a recombination hotspot is insufficient to increase crossover frequency in Arabidopsis
Source: G3 (Bethesda). 2022 Apr 29;12(7):jkac105. doi: 10.1093/g3journal/jkac105 (PMC9258527; doi:10.1093/g3journal/jkac105)
Supplement: jkac105_Supplementary_Figure_Legends [file jkac105_supplementary_figure_legends.docx]

**SUPPLEMENTAL FIGURE LEGENDS**

**Supplemental Figures S1-S2. Sanger sequencing analysis of gene editing events in *3a* crossover hotspot.** Nucleotide sequences of each of the target regions (*3a-P*, *3a-B* and *3a-I*) are shown, gRNA target sites highlighted in grey and protospacer adjacent motif, PAM, in red. To distinguish between overlapping gRNA-B-1 and gRNA-B-2, in addition to highlighting them in grey, one is shown bold and the other is underlined. Dots indicate wild type genomic sequence not shown due to space constraints, dashes indicate sequences deleted via CRISPR/Cas9-mediated gene editing. In the case where CRISPR/Cas9 editing causes an 8 bp insertion, dashes indicate a corresponding missing sequence in the wild type.

**Supplemental Figure S3. Testing the efficiency of guide RNAs targeting outside *3a* crossover hotspot. (A)** Schematic representation of the five Arabidopsis chromosomes and gRNA positions. Chromosomes - green bars, blue rectangles - centromeres, red ticks - genes targeted with CRISPR/Cas9, star - 3a hotspot (shown for reference). **(B)** Constructs harbouring guide RNAs targeting six Arabidopsis genes (*CLE9*, *CLE10*, *CLV3*, *GL1*, *FWA* and *eIF(iso)4E*)) and a catalytically active Cas9 were transformed into wild type Col. T1 progenies were selected for the presence of *gRNA-Cas9* transgenes and tested for gene editing events. Representative Midori-green-stained agarose gel analyses of CRISPR/Cas9-mediated gene editing in T1 leaf tissue. Mutations introduced by CRISPR/Cas9 either destroy a restriction endonuclease recognition site (*Bsu36*I in *CLE10*, *BspH*I in *CLV3* and *Dde*I in *GL1*) resulting in higher molecular weight products compared to wild type control (untransformed Col) or introduce a mismatch that is recognised by T7 Endonuclease I resulting in endonucleolytic cleavage and lower molecular weight product(s) compared to untransformed Col control (in *CLE9*, *eIF(iso)4E* and *FWA*). Different leaf sectors of the analysed T1 plants were mosaic for CRISPR/Cas9-mediated gene editing events and had one (‘het’), two (‘ko) or neither (‘wt’) gene edited allele(s). This was visually demonstrated for *GL1* responsible for trichome development. Leaf segments showing absence of trichomes (white arrows) had both *GL1* alleles mutated by CRISRP/Cas9. **(C)** Summary table showing number and percentage of T1s affected by CRISPR/Cas9 gene editing.

**Supplemental Figure S4. Detection of mature gRNAs via RT-PCR.** (A) Schematic representation of a mature gRNA. An (RT-)PCR assay to detect mature gRNAs uses a ‘universal’ reverse primer complementary to the 3’ end of the gRNA scaffold (black arrow) and a guide-RNA-spacer-specific forward primer, light blue arrow. (B) Detection of one of the six mature gRNAs targeting 3a-P by RT-PCR in Col/Ws mtopvib MTOPVIB-dCas9 gRNA-P F1 population. Black arrow points to the mature gRNA-specific PCR product resolved on a Midori-green-strained 2% agarose gel. Col and ‘no gRNA’ are negative controls. Two biological and two technical replicates were done for each genotype. Higher than 96-bp molecular weight bands are non-specific products. (C) As in (B) but for gRNA-B. (D) As in (B) but for gRNA-I. Two to three out of the six multiplexed gRNAs for each of the target regions was tested, but data for one gRNA is shown. (E) Ubiquitin was used as a ‘+RT’ and ‘-RT’ controls.

**Supplemental Figure S5. ChIP-qPCR analysis of MTOPVIB-dCas9 enrichment at 3a crossover hotspot.** Chromatin IP was performed with an antibody against the HA epitope tag using 10 grams of closed flower buds as starting material for each of the Col/Ws F_2_ populations selected to express *MTOPVIB-2×HA-dCas9* and either no guide RNAs or *gRNA-P*, *gRNA-B* or *gRNA-I* targeting *3a*. Wild type Col was used as a control. *y*-axis represents enrichment normalized to input and relative to Col. At3g2875 is a region outside 3a and is used as a control. Two different PCR primer pairs were used for amplicons within *3a-B* and *3a-I*.
